# Panitumumab Plus Trifluridine-Tipiracil as Anti–Epidermal Growth Factor Receptor Rechallenge Therapy for Refractory *RAS* Wild-Type Metastatic Colorectal Cancer: A Phase 2 Randomized Clinical Trial

**DOI:** 10.1001/jamaoncol.2023.0655

**Published:** 2023-05-18

**Authors:** Stefania Napolitano, Vincenzo De Falco, Giulia Martini, Davide Ciardiello, Erika Martinelli, Carminia Maria Della Corte, Lucia Esposito, Vincenzo Famiglietti, Alessandra Di Liello, Antonio Avallone, Claudia Cardone, Alfonso De Stefano, Vincenzo Montesarchio, Maria Giulia Zampino, Roberto Bordonaro, Mario Scartozzi, Daniele Santini, Massimo Di Maio, Ferdinando De Vita, Lucia Altucci, Francesca Marrone, Fortunato Ciardiello, Teresa Troiani

**Affiliations:** 1 Department of Precision Medicine, Università Degli Studi Della Campania “Luigi Vanvitelli,” Napoli, Italy; 2 Medical Oncology, Fondazione IRCCS Casa Sollievo Della Sofferenza, San Giovanni Rotondo, Italy; 3 Oncologia Medica, Istituto Nazionale per lo Studio e la Cura dei Tumori “Fondazione Giovanni Pascale”—IRCCS, Napoli, Italy; 4 UOC Oncologia, AORN dei Colli (Monaldi-Cotugno-CTO), Napoli, Italy; 5 Unit of Gastrointestinal and Neuroendocrine Tumors, Division of Medical Oncology, European Institute of Oncology, Milano, Italy; 6 Medical Oncology Unit, ARNAS Garibaldi, Catania, Italy; 7 Medical Oncology, University and University Hospital of Cagliari, Cagliari, Italy; 8 Oncologia Medica, Campus Biomedico, Roma, Italy; 9 Department of Oncology, University of Turin at Ordine Mauriziano Hospital, Turin, Italy

## Abstract

**Importance:**

Current third-line therapies for patients with metastatic colorectal cancer (MCRC) have limited efficacy. Rechallenge with epidermal growth factor receptor (EGFR) inhibitors for *RAS* wild-type (WT) MCRC may be valuable for these patients.

**Objective:**

To compare the anti-EGFR monoclonal antibody panitumumab plus standard-of-care trifluridine-tipiracil with trifluridine-tipiracil alone as third-line therapy for *RAS* WT MCRC.

**Design, Setting, and Participants:**

This phase 2 randomized clinical trial (RCT) was conducted in 7 Italian centers from June 2019 to April 2022. Patients with refractory *RAS* WT MCRC who had a partial or complete response to first-line chemotherapy plus an anti-EGFR monoclonal antibody and an anti-EGFR drug–free interval of 4 or more months during second-line therapy were included.

**Interventions:**

Patients were randomized 1:1 to receive panitumumab plus trifluridine-tipiracil or trifluridine-tipiracil alone.

**Main Outcomes and Measures:**

The primary end point was progression-free survival (PFS). Circulating tumor DNA (ctDNA) extended sequence variation analysis was performed in a subgroup of patients.

**Results:**

Of 62 included patients, 31 received panitumumab plus trifluridine-tipiracil (19 [61.3%] male; median age, 65 years [range, 39-81 years]) and 31 received trifluridine-tipiracil alone (17 [54.8%] male; median age, 66 years [range, 32-82 years]). The primary end point was met. Median PFS was 4.0 months (95% CI, 2.8-5.3 months) in the panitumumab plus trifluridine-tipiracil arm vs 2.5 months (95% CI, 1.4-3.6 months) in the trifluridine-tipiracil only (hazard ratio [HR], 0.48; 95% CI, 0.28-0.82; *P* = .007). Pretreatment plasma *RAS/BRAF* WT ctDNA identified patients obtaining prolonged clinical benefit with panitumumab plus trifluridine-tipiracil compared with trifluridine-tipiracil, with PFS rates at 6 months of 38.5% vs 13.0% and at 12 months of 15.4% vs 0%. A ctDNA liquid-biopsy extended mutation analysis by FoundationOne Liquid CDx (profiling 324 genes) was performed in a subgroup of patients with baseline plasma *RAS/BRAF* WT ctDNA; in 15 of 23 patients (65.2%) whose tumors were WT for *KRAS*, *NRAS*, *BRAFV600E*, *EGFR*, *ERBB2*, *MAP2K1*, and *PIK3CA*, median PFS was 6.4 months (95% CI, 3.7-9.2 months). Within this group of 15 patients, 2 (13.3%) had partial response, 11 (73.3%) had stable disease, and 2 (13.3%) had disease progression as best response.

**Conclusions and Relevance:**

In this RCT, third-line treatment with the anti-EGFR monoclonal antibody panitumumab plus the standard-of-care trifluridine-tipiracil resulted in improved PFS compared with treatment with trifluridine-tipiracil alone among patients with refractory *RAS* WT MCRC. The findings support the clinical utility of liquid biopsy–guided anti-EGFR rechallenge therapy for refractory *RAS* WT MCRC.

**Trial Registration:**

ClinicalTrials.gov Identifier: NCT05468892

## Key Points

**Question:**

Does an anti–epidermal growth factor receptor rechallenge approach for *RAS* wild-type metastatic colorectal cancer (MCRC) using panitumumab plus trifluridine-tipiracil lead to improved progression-free survival (PFS) compared with the standard-of-care trifluridine-tipiracil?

**Findings:**

In this randomized clinical trial of 62 Italian patients with refractory *RAS* wild-type MCRC, panitumumab plus trifluridine-tipiracil increased PFS at 6 and 12 months compared with trifluridine-tipiracil alone.

**Meaning:**

The findings suggest that PFS is improved with panitumumab plus trifluridine-tipiracil compared with trifluridine-tipiracil alone in patients with *RAS* wild-type MCRC.

## Teaser

This randomized clinical trial assesses progression-free survival among patients with *RAS* wild-type metastatic colorectal cancer receiving panitumumab plus standard-of-care trifluridine-tipiracil as third-line therapy.

## Introduction

Anti–epidermal growth factor receptor (EGFR) monoclonal antibodies plus cytotoxic drugs are the treatment cornerstone for *RAS* wild-type (WT) metastatic colorectal cancer (MCRC).^1,2^ Despite initial efficacy, acquired resistance mechanisms, including *KRAS* or *NRAS* alterations,^3,4,5^ are associated with treatment failure in all patients.^3,4^ Whereas the majority of *RAS* WT cancer cells are killed by chemotherapy plus cetuximab or panitumumab, a genetic selection of *RAS* mutant cancer cells occurs with tumor progression. A subsequent treatment, such as chemotherapy plus antiangiogenic drugs, could cause the disappearance of *RAS* mutant clones and potentially restore sensitivity to anti-EGFR drugs.^6^ After anti-EGFR treatment is stopped, *RAS* mutant clones decay with a half-life of approximately 4 months, whereas *RAS* WT clones increase.^7^ In fact, the genetic landscape of cancer cell clones is continuously evolving for the selective pressure of different therapies.^8^

Regorafenib or trifluridine-tipiracil is used after failure of 2 lines of therapy,^1,2^ with modest benefit in overall survival and progression-free survival (PFS).^1,2^ Therefore, anti-EGFR rechallenge therapy has been proposed for patients with *RAS* WT who have experienced treatment failure.^3,6^ Three single-arm phase 2 trials have supported anti-EGFR rechallenge therapies.^9,10,11^ However, important questions remain, including the best anti-EGFR rechallenge approach and how anti-EGFR rechallenge compares with the standard of care.^6^ We conducted VELO, a phase 2 randomized clinical trial (RCT), to determine the contribution of panitumumab to standard-of-care trifluridine-tipiracil as a third-line therapy in patients with *RAS* WT MCRC.

## Methods

This RCT (NCT05468892) was conducted at 7 Italian centers from June 2019 to April 2022. The VELO trial protocol is in Supplement 1. The trial was approved by the institutional review board of Università Degli Studi Della Campania Luigi Vanvitelli–Azienda Ospedaliera Universitaria Luigi Vanvitelli–AORN Ospedale dei Colli. The protocol was approved by the local ethics committees of the participating sites. All patients provided written informed consent. We followed the Consolidated Standards of Reporting Trials (CONSORT) reporting guideline.

The trial included patients with refractory *RAS* WT MCRC who had a partial response (PR) or complete response to first-line chemotherapy plus an anti-EGFR monoclonal antibody and an anti-EGFR drug–free interval of 4 or more months during second-line therapy. Patients were randomized 1:1 to receive panitumumab plus trifluridine-tipiracil or trifluridine-tipiracil alone. The random allocation sequence was generated by an external data manager with no clinical involvement in the study. The study was open label, and treatment assignment was not masked to the patients or the investigators. The primary end point (prespecified) was PFS, defined as the time from randomization to the earliest documented disease progression or death due to any cause. Secondary end points (prespecified) were objective response rate, incidence of adverse events, overall response rate, and overall survival. An exploratory analysis included genomic analysis by next-generation sequencing (NGS) of plasma samples collected at baseline and, if possible, at the end of treatment (eMethods in Supplement 2). Blood samples were analyzed for plasma circulating tumor DNA (ctDNA) by the Idylla Biocartis platform and FoundationOne Liquid CDx.^12,13^ All patients were assessed before rechallenge treatment by the Idylla Biocartis platform to detect known resistance alterations in *KRAS* (exons 2, 3, and 4), *NRAS* (exons 2, 3, and 4), and *BRAFV600E*.^12^ Pretreatment plasma samples from 46 patients were analyzed by NGS (profiling 324 genes) with FoundationOne Liquid CDx.^13^ For 24 patients, plasma samples were also analyzed at disease progression with FoundationOne Liquid CDx.

### Statistical Analysis

Distributions of time-to-event variables were estimated with the use of the Kaplan-Meier product-limit method. The stratified log-rank test was used as the primary analysis for comparison of treatment groups. Hazard ratios (HRs) and 95% CIs were estimated with the Cox proportional hazards regression model. More details of the statistical analysis are given in the eMethods in Supplement 2. Analyses were performed using IBM SPSS, version 23.0 (IBM). Significance was set at 2-sided *P* < .05.

## Results

**Figure 1.**
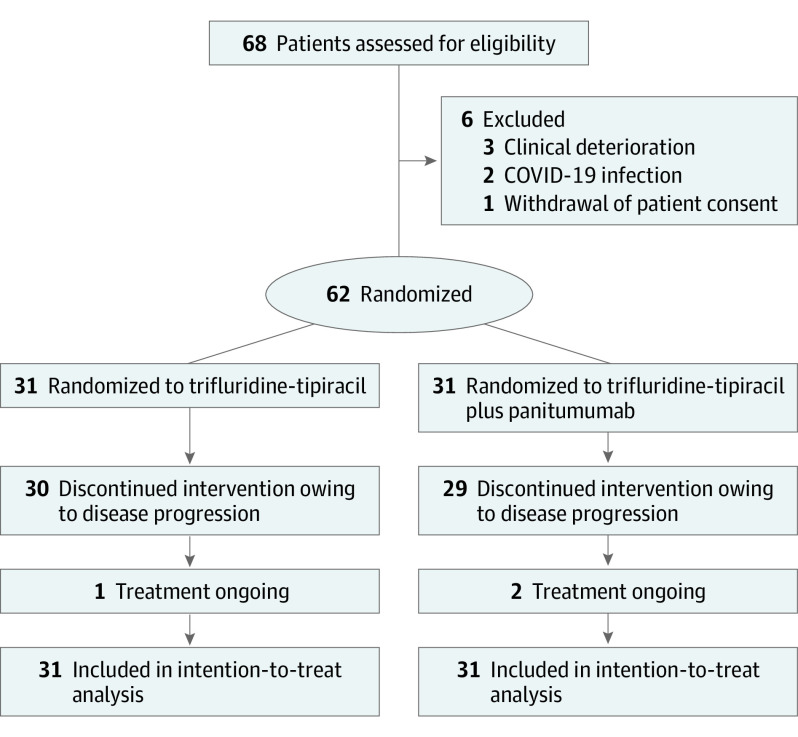
CONSORT Diagram The data cutoff for the interim analysis was September 16, 2022.

Of 62 patients included in the trial, 31 received panitumumab plus trifluridine-tipiracil (19 [61.3%] male; median age, 65 years [range, 39-81 years]) (arm B) and 31 received trifluridine-tipiracil only (17 [54.8%] male; median age, 66 years [range, 32-82 years]) (arm A) (Figure 1). Disease characteristics were balanced between the groups. Before rechallenge treatment, liquid biopsy was performed by Idylla Biocartis platform.^4,12^ No *BRAFV600E* alterations were found in the patients; *RAS/BRAF* WT ctDNA was found in 23 patients (74.2%) in arm A and in 26 (83.9%) in arm B (eTable 1 in Supplement 2).

The median number of treatment cycles was 4 (range, 1-26) in arm B and 2 (range, 1-10) in arm A (eTable 2 in Supplement 2). Grade 3 and 4 adverse events occurred in 16 patients in arm B (51.6%) and 9 patients in arm A (29.0%) (eTable 3 in Supplement 2). There were no treatment-related deaths and no treatment discontinuation due to adverse events. Dose reductions were necessary in 16 patients in arm B (51.6%) and in 9 patients in arm A (29.0%) (*P* = .07) (eTable 2 in Supplement 2).

**Figure 2.**
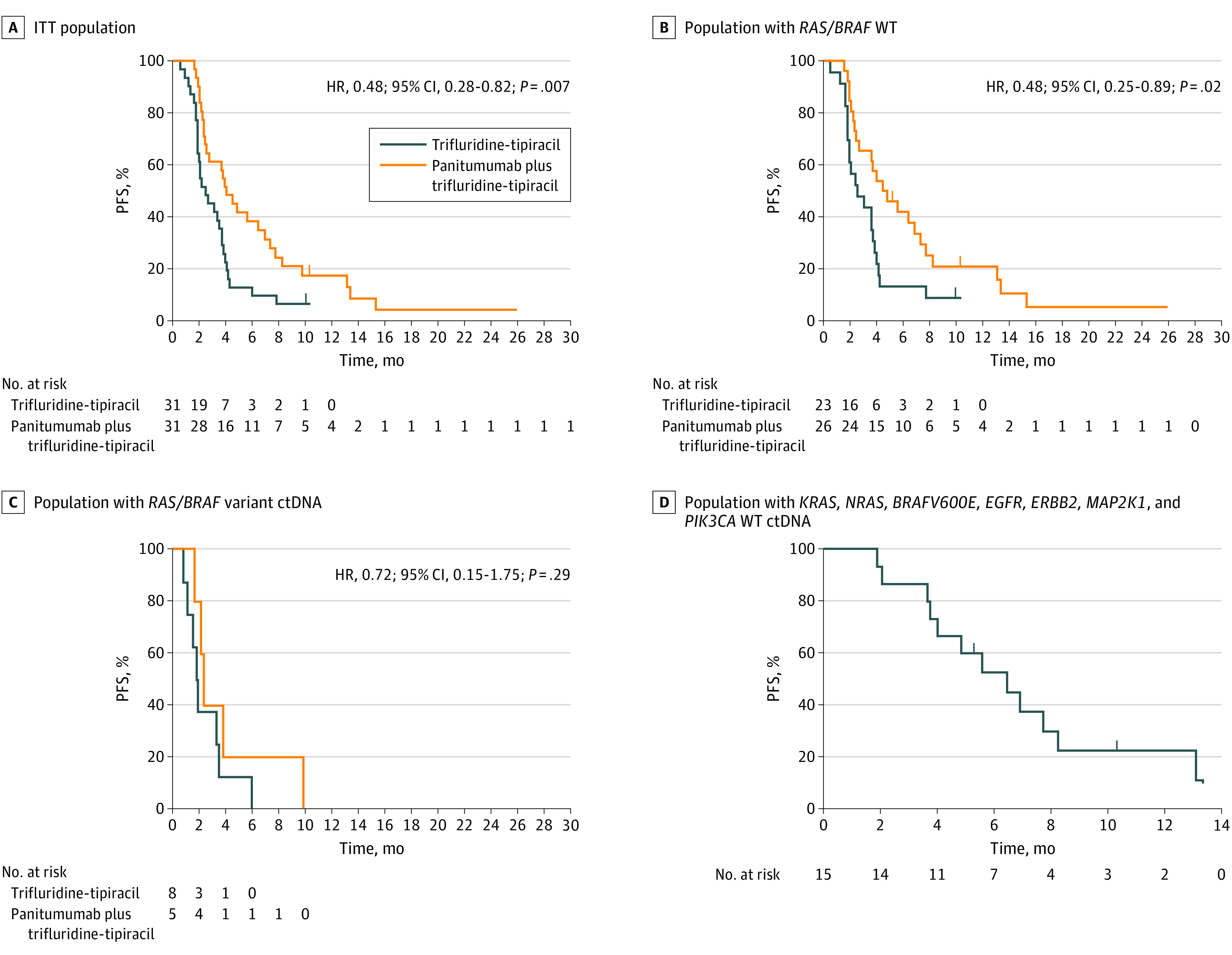
Kaplan-Meier Estimates of Progression-Free Survival (PFS) in the Study Population Cross marks show data censored at the time of last disease assessment. ctDNA indicates circulating tumor DNA; ITT, intention to treat; and WT, wild type.

The primary end point was met. Treatment with panitumumab plus trifluridine-tipiracil resulted in longer PFS, with 52% reduction in the risk of progression (HR, 0.48; 95% CI, 0.28-0.82; *P* = .007). Median PFS was 2.5 months (95% CI, 1.4-3.6 months) in arm A and 4.0 months (95% CI, 2.8-5.3 months) in arm B (Figure 2A). Confirmed PR only occurred in arm B (3 patients [9.7%]). Disease control rates of 4 months or more (calculated as PR plus complete response plus stable disease) were 74.2% in arm B and 38.7% in arm A (*P* = .009). The PFS rates at 6 months and 12 months were higher in arm B than in arm A (6 months: 35.5% vs 9.7% [*P* = .02]; 12 months: 12.9% vs 0% [*P* = .04]) (eFigure 1 in Supplement 2).

In patients with pretreatment plasma *RAS/BRAF* WT ctDNA, median PFS was 4.5 months (95% CI, 2.2-6.8 months) in arm B vs 2.6 months (95% CI, 1.0-4.3 months) in arm A (HR, 0.48; 95% CI, 0.26-0.89; *P* = .02) (Figure 2B). The disease control rate was 80.7% in patients with pretreatment plasma *RAS/BRAF* ctDNA WT in arm B vs 47.8% in arm A (eFigure 1 in Supplement 2). The PFS rates were also higher in patients with pretreatment plasma ctDNA *RAS/BRAF* WT in arm B compared with arm A (6 months: 38.5% vs 13.0% [*P* = .047]; 12 months: 15.4% vs 0% [*P* = .052]), but the difference at 12 months was not significant (eFigure 1 in Supplement 2). In patients with pretreatment plasma *RAS* variant ctDNA, no advantage by adding panitumumab to trifluridine-tipiracil was observed (HR, 0.72; 95% CI, 0.15-1.75; *P* = .29) (Figure 2C).

Before rechallenge treatment, NGS liquid biopsy findings were available for 16 of 23 patients (69.6%) with *RAS/BRAF* WT ctDNA in arm A and for 23 of 26 patients (88.5%) with *RAS/BRAF* WT ctDNA in arm B (eFigure 2 in Supplement 2). The NGS analysis found that *KRAS*, *PIK3CA*, *BRAF*, *MAP2K1*, and *EGFR* were the most frequently mutated genes within the EGFR pathway, whereas *TP53*, *APC*, *ARID1A*, and *SMAD4* were the most frequently mutated tumor suppressor genes (eFigure 3 in Supplement 2 and eTable 4 in Supplement 3). Alterations in *APC* and *TP53* were found in almost all patient samples (43 of 46 [93.5%] and 42 of 46 [91.3%], respectively).

For arm B, in 15 of 23 patients whose tumors were WT for *KRAS*, *NRAS*, *BRAFV600E*, *EGFR*, *ERBB2*, *MAP2K1*, and *PIK3CA* (65.2%), median PFS was 6.4 months (95% CI, 3.7-9.2 months) (Figure 2D). Of those 15 patients, 2 (13.3%) had PR, 11 (73.3%) had stable disease, and only 2 (13.3%) had disease progression. In 6 of 11 patients with *RAS/BRAF* WT ctDNA in arm B for whom FoundationOne Liquid CDx findings were available both at pretreatment and at disease progression (54.5%) (eFigure 4 in Supplement 2 and eTable 5 in Supplement 3), 1 or more sequence variations in the EGFR pathway were found at progression.

## Discussion

To our knowledge, the VELO trial is the first phase 2 RCT in which the combination of an anti-EGFR drug (panitumumab) with a standard-of-care drug (trifluridine-tipiracil) has been evaluated as anti-EGFR rechallenge therapy for refractory *RAS* WT MCRC. The study met the primary end point. The risk of disease progression was reduced by 52%, and the disease control rate was almost doubled (74.2% vs 38.7%). With the limitations of a small trial, we provided evidence of improved clinical activity of anti-EGFR rechallenge therapy compared with the standard of care.

These findings also support the use of pretreatment plasma ctDNA for patient selection.^6,9,10,11^ Patients with plasma *RAS/BRAF* WT ctDNA before rechallenge who were treated with panitumumab plus trifluridine-tipiracil had better outcomes in terms of median PFS, reduced risk of progression, 6- and 12-month probability of being without progression, and objective responses compared with those without pretreatment plasma *RAS/BRAF* WT ctDNA.

Whereas the polymerase chain reaction–based Idylla Biocartis platform captured only known alterations for *KRAS* and *NRAS* as well as the *BRAFV600E* alteration, NGS-based 324 genomic profiling of plasma ctDNA by FoundationOne Liquid CDx^12,13^ identified additional alterations and amplifications in EGFR pathway genes that correlated with lack of activity. In 15 patients with EGFR pathway WT genes, median PFS reached 6.4 months. Within the limitation of a subgroup analysis, these results suggest that NGS-based assessment of plasma ctDNA could allow a more precise selection for anti-EGFR rechallenge therapies. The NGS analysis was also done before treatment and at disease progression. The emergence of cancer cell resistance due to EGFR pathway alterations was found in the majority of patients who received panitumumab plus trifluridine-tipiracil.

### Limitations

This study has limitations. The main limitation is the small size of the subgroup analysis, which was severely affected by the COVID-19 pandemic. Larger, phase 3 randomized clinical trials are needed to confirm the clinical efficacy of this approach.

## Conclusions

In this RCT, third-line treatment with the anti-EGFR monoclonal antibody panitumumab plus the standard-of-care trifluridine-tipiracil resulted in improved PFS compared with treatment with trifluridine-tipiracil alone among patients with refractory *RAS* WT MCRC. Several anti-EGFR rechallenge trials are ongoing.^14,15^ Although these studies will define the most effective regimens, the VELO trial supports the clinical benefit of liquid biopsy–guided anti-EGFR rechallenge therapy.

## Notes

Supplement 1.
Trial Protocol

Supplement 2. **eMethods.** Outcomes and Statistical Analysis **eTable 1.** Baseline Demographic and Disease Characteristics for the ITT Population at the Time of Randomization **eTable 2.** Treatment-Emergent Adverse Events **eTable 3.** Treatment-Emergent Adverse Events by Grade **eFigure 1.** Best Response in Study Population **eFigure 2.** Molecular Screening of the VELO Trial **eFigure 3.** Correlation of Molecular Alterations Detected on ctDNA by FoundationOne CDx Liquid Analysis With Clinical Data **eFigure 4.** Landscape of Molecular Alterations Detected by FoundationOne CDx Liquid Analysis in 24 Patients With RAS/BRAF WT at Baseline and at Progression of Disease

Supplement 3. **eTable 4.** FoundationOne CDx Liquid Analysis on ctDNA of 46 Evaluable Patients at Baseline Rechallenge **eTable 5.** FoundationOne CDx Liquid Analysis on ctDNA of 24 Patients With RAS/BRAF WT at Baseline Rechallenge and at Disease Progression

Supplement 4.
Data Sharing Statement
