# Supplementary material for: Panitumumab Plus Trifluridine-Tipiracil as Anti–Epidermal Growth Factor Receptor Rechallenge Therapy for Refractory RAS Wild-Type Metastatic Colorectal Cancer: A Phase 2 Randomized Clinical Trial
Source: JAMA Oncol. 2023 May 18;9(7):966–70. doi: 10.1001/jamaoncol.2023.0655 (PMC10196928; doi:10.1001/jamaoncol.2023.0655)
Supplement: Supplement 1. — Trial Protocol [file jamaoncol-e230655-s001.pdf]

CLINICAL STUDY PROTOCOL

VELO

Phase II randomized study evaluating the efficacy of panitumumab (VEctibix) and Trifluridine-Tipiracil (LOnsurf) in pretreated *RAS* wild type metastatic colorectal cancer patients: the VELO trial

**Protocol code:**	**VELO**
**Protocol version:**	2.0
**Protocol date:**	10/Apr/2019
**EudraCT number:**	
**Sponsor:**	Dipartimento di Medicina di Precisione, Università degli studi della Campania Luigi Vanvitelli.

TABLE OF CONTENTS

1 SYNOPSIS 8
1.1 BACKGROUND 11
1.2 STUDY RATIONALE 12
2 AIMS OF THE STUDY 14
2.1 Study Hypothesis 14
2.2 Objectives of the study 14
2.2.1 Primary objective 14
2.2.2 Secondary objectives 14
2.2.3 Exploratory secondary objectives 14
2.3 Endpoints of the study 14
2.3.1 Primary Endpoint 14
2.3.2 Secondary Endpoints 15
2.3.3 Exploratory secondary Endpoints 15
3 SELECTION OF PATIENTS 15
3.1 Inclusion Criteria 15
3.2 Exclusion Criteria 17
4 STUDY DESIGN AND PROCEDURES 18
4.1 Sample size 19
4.2 Study duration 19
4.3 Treatment plan 19
4.3.1 Treatment dosage and schedule 20
4.3.2 Dose modification, delays and interruptions criteria 23
4.3.3 Concomitant treatments 29
4.4 Schedule of assessments and procedures 31
4.4.1 Procedures for enrolment of eligible patients 31
4.4.2 Screening and Baseline Assessments 32
4.4.3 Assessment during treatment 33
4.4.4 Assessments at End of Treatment 34
4.4.5 Follow-Up Assessments 34
4.4.6 Collection of CT scans 35
5. RESPONSE EVALUATION 39
5.1 Measurability of tumor lesions 39
5.2. Identification of "target" and "non-target" lesions 39
5.3 Evaluation of target lesion response 40
5.4. Evaluation of non-target lesion response 40
5.5. Evaluation of best overall response 40
5.6. Reporting of results 41
6. BIOMARKERS 41
6.1 Blood Sample Collection and analysis 41
6.2 Tumor Tissue Samples Collection 41
7. SAFETY INSTRUCTIONS AND GUIDANCE 42
7.1 Warning and management of specific toxicities 42
7.2 Adverse Events and Laboratory Abnormalities 44
7.2.1 Clinical Adverse Events (AEs) 44
7.2.2 Adverse Drug Reaction (ADR) 46

7.2.3 Serious Adverse Events (SAE) 46
7.2.4 Serious adverse drug reaction (SADR)........ 47
7.2.5 Special situations........ 48
7.3 Treatment and Follow-up of AEs 49
7.4 Laboratory Test abnormalities 49
7.4.1 Follow-up of Abnormal Laboratory Test Values 50
7.5 Handling of Safety Parameters........ 50
7.5.1 Reporting of AEs........ 50
7.5.2 Reporting of SAEs (immediately reportable) 50
7.5.3 Pregnancy 51
7.5.4 Safety data exchange requirement 52
8. STATISTICAL CONSIDERATIONS........ 52
8.1 Sample size calculation 52
8.2 Analysis Populations 53
8.3 Statistical analysis 53
9. DATA COLLECTION AND MANAGEMENT 55
9.1 Data confidentiality........ 55
9.2 Site monitoring........ 55
9.3 Data collection........ 55
9.4 Database management and quality control 56
10. ETHICAL CONSIDERATION 56
10.1 Regulatory and ethical compliance 56
10.2 Responsibilities of the investigator and IEC 56
10.3 Informed consent procedures 56
10.4 Publication of study protocol and results 56
10.5 Study documentation, record keeping and retention of documents 57
10.6 Confidentiality of study documents and patient records 57
10.7 Audits and inspections 57
10.8 Financial disclosures 58
11. PROTOCOL ADHERENCE 58
11.1 Amendments to the protocol 58
12. ADMINISTRATIVE ASPECTS 58
12. 1 Investigational medical product 58
12.1.1 Formulation, Packaging and Labelling 58
12.1.2 Accountability, assessment of compliance and destruction of the drugs........ 59
13. References 60
14 APPENDICES 63
....

LIST OF ABBREVIATIONS

AE Adverse Event
ANC Absolute neutrophil count
CRF Case Report Form
CI Confidence interval
CNS Central nervous system
CRC Colorectal cancer
CTC Common toxicity criteria
cfDNA Circulating free tumor DNA
ECOG Eastern Cooperative Oncology Group
ECG Electrocardiogram
EGFR Epidermal Growth Factor Receptor
GCP Good Clinical Practice
HR Hazard ratio
ICH International Conference on Harmonization
IEC Independent Ethics Committee
IRB Institutional Review Board
ITT Intention To Treat
mCRC Metastatic colorectal cancer
MoAb Monoclonal antibody
NGS Next generation sequencing
OR Odds ratio
ORR Overall response rate
OS Overall survival
PD Disease progression
PFS Progression free survival
Pts Patients
PS Performance status
QoL Quality of life
RR Response rate
SAE Serious Adverse Event
SC Steering Committee
WBC white blood cell
WT Wild type

1 PROTOCOL SYNOPSIS

**Study Title**	Phase II randomized study evaluating the efficacy of panitumumab (VEctibix) and Trifluridine-Tipiracil (LOnsurf) in pretreated *RAS* wild type metastatic colorectal cancer patients:<br>the VELO trial
**Study ID**	
**Study Acronyms**	VELO
**EudraCT Number**	2018-001600-12
**Trial Registration ClinicalTrials.Gov Number**	NCT XXXXX
**Sponsor**	Dipartimento di Medicina di Precisione,<br>Università degli studi della Campania "Luigi Vanvitelli"
**Setting**	Patients with *RAS* WT metastatic colorectal cancer, third line setting
**Phase**	II
**Number of patients**	112
**Background**	The Epidermal Growth Factor Receptor (EGFR) targeted therapy with the monoclonal antibodies cetuximab or panitumumab represents a major step forward in the treatment of *RAS* wild type (WT) metastatic colorectal cancer (mCRC), given the relevant efficacy in terms of progression-free survival (PFS), overall survival (OS), response rate (RR), as well as quality of life (QoL), observed in several phase III clinical trials among different lines of treatment. However, the clinical benefit observed with these agents is limited to only a subset of patients and responses are often transient due to the development of various mechanisms of resistance. Several studies have provided new insights into molecular basis of EGFR inhibitors resistance and have identified mutations in KRAS, NRAS, BRAF and EGFR extracellular domain (ECD) as well as the amplification of ERBB2 and MET, as biomarkers of both primary and/or acquired resistance to these drugs. Unraveling the biology underlying the complex mechanisms of resistance have been useful for developing rational combination therapies in order to revert or overcome resistance. Rechallenge with an alternative anti-EGFR monoclonal antibody (MoAb) after failure with an agent of the same family has been proposed as strategy to overcome drug resistance. However, panitumumab, as single agent, has demonstrated to provide minimal benefit in patients with KRAS WT mCRC who have experienced progression to cetuximab as prior therapy. The hypotheses that pre-existing sensitive subclones may emerge after treatment breaks with anti-EGFR MoAb has led the design of several clinical trials prospectively evaluating the rechallenge with anti-EGFR MoAbs in the third-line setting after a response to a first-line therapy with anti-EGFR drugs. Trifluridine-Tipiracil demonstrated to be effective in the treatment of refractory mCRC. Anti-EGFR agents in combination with Trifluridine-

	Tipiracil have shown preliminary activity in SW48 (KRAS WT) xenograft models. Panitumumab in combination with Trifluridine-Tipiracil could be a valid therapeutic option for mCRC *RAS* WT patients that achieved a major response in the first line of therapy to panitumumab or cetuximab as third line rechallenge treatment.
**Rationale**	The aim of this study is to investigate a rechallenge strategy of panitumumab in combination with Trifluridine-Tipiracil as compared to standard third line therapy (Trifluridine-Tipiracil) in patients with *RAS* WT mCRC treated in first line with chemotherapy in combination with an anti-EGFR drug that have had a clinical benefit (complete or partial response) from treatment.
**Primary Objectives**	To explore the efficacy of panitumumab in combination with Trifluridine-Tipiracil vs standard third line therapy (Trifluridine-Tipiracil) as measured by PFS
**Secondary Objectives**	To compare the activity of panitumumab in combination with Trifluridine-Tipiracil vs standard third line therapy (Trifluridine-Tipiracil) as measured by ORR<br>To compare the efficacy of panitumumab in combination with Trifluridine-Tipiracil vs standard third line therapy (Trifluridine-Tipiracil) as measured by OS<br>To explore the safety and tolerability of panitumumab in combination with Trifluridine-Tipiracil vs standard third line therapy (Trifluridine-Tipiracil)
**Study plan**	This is a randomized phase II, open label, two arm study, evaluating the efficacy of panitumumab in combination with Trifluridine-Tipiracil as third line therapy, after a first line containing an anti-EGFR agent panitumumab (at least 70% of study population) or cetuximab.<br><br>Arm A: Trifluridine-Tipiracil<br>Arm B: Panitumumab+ Trifluridine-Tipiracil
**Sample size**	The study is designed to have 80% power to detect a hazard ratio for progression of 0.56 (a 44% reduction in risk) in the Trifluridine-Tipiracil + panitumumab group as compared with the Trifluridine-Tipiracil group, with a two-sided type I error rate of 0.1. Given the treatment assignment ratio of 1:1 (Trifluridine-Tipiracil : panitumumab + Trifluridine-Tipiracil), we calculate that at least 74 events (progression) in 112 subjects (56 in the control group and 56 in the treatment group) would be required for the primary analysis.
**Inclusion criteria**	• Histologically proven diagnosis of colorectal adenocarcinoma<br>• Diagnosis of metastatic disease<br>• *RAS* (NRAS and KRAS exon 2,3 and 4) wild-type in tissue at initial diagnosis<br>• Efficacy of a first line therapy containing an anti-EGFR agent (panitumumab or cetuximab) with a major response achieved (complete or partial response)

	• Progression after a second line therapy<br>• Available and adequate baseline tumour tissue sample<br>• Measurable disease according to RECIST criteria v1.1<br>• Male or female patients > 18 years of age<br>• ECOG Performance Status 0-1<br>• Life expectancy of at least 3 months<br>• Adequate bone marrow, liver and renal function<br>• If female and of childbearing potential, have a negative result on a pregnancy test performed a maximum of 14 days before initiation of study treatment<br>• If female and of childbearing potential, or if male, agreement to use adequate contraception (eg, abstinence, intrauterine device, oral contraceptive, or double-barrier method)<br>• Signed informed consent
**Exclusion criteria**	• Any contraindication to use Trifluridine - Tipiracil or Panitumumab<br>• More than two previous lines of treatment<br>• Active uncontrolled infections<br>• Past or current history of malignancies other than colorectal carcinoma, except for curatively treated basal and squamous cell carcinoma of the skin or in situ carcinoma of the cervix<br>• Pregnancy<br>• Breastfeeding<br>• Interstitial lung disease or pulmonary fibrosis<br>• Grade III or IV heart failure (NYHA classification)
**Statistical methods**	The study is designed to have 80% power to detect a hazard ratio for progression of 0.56 (a 44% reduction in risk) in the Trifluridine-Tipiracil + panitumumab group as compared with the Trifluridine-Tipiracil group, with a two-sided type I error rate of 0.1. Given the treatment assignment ratio of 1:1 (Trifluridine - Tipiracil: Panitumumab + Trifluridine - Tipiracil), we calculate that at least 74 events (progression) in 112 subjects (56 in the control group and 56 in the treatment group) would be required for the primary analysis.<br><br>The median period of follow-up will be calculated for the entire study cohort according to the reverse Kaplan–Meier method. Distributions of time-to-event variables will be estimated with the use of the Kaplan–Meier product-limit method. The stratified log-rank test will be used as the primary analysis for comparison of treatment groups. Cox proportional-hazards modeling will be also performed as supportive analyses. Subgroup analyses of progression-free survival will be performed by means of an interaction test to determine the consistency of the treatment effect according to key baseline characteristics. Overall survival will be analyzed with the same methods used for the analysis of progression-free survival.<br>The objective response rate and the incidence of adverse events in the different groups will be compared with the use of the chi-square test for heterogeneity.<br>All statistical tests will be two-sided, and P values of 0.05 or less will be considered to indicate statistical significance. Odds ratios and 95%

	confidence intervals will be estimated with a logistic-regression model, and hazard ratios and 95% confidence intervals will be estimated with a Cox proportional hazards model.
**Duration of the study**	30 months
**Total Number of Centers**	13 Italian centers
**Data Management**	Mrs. Daniela Renato<br>Dr Vincenzo Famiglietti
**Contact for Scientific Issues**	Prof Fortunato Ciardiello<br>Dr Erika Martinelli<br>Dr Teresa Troiani<br>Dr Claudia Cardone<br>Dr Vincenzo De Falco
**Contact for Coordination and Administrative Issues**	Mrs Daniela Renato

1.1 BACKGROUND

Colorectal cancer (CRC) is the second most commonly diagnosed cancer in Europe and a leading cause of cancer death in the world, accounting for about 1.4 million new cases and almost 700.000 deaths in 2012 (Ferlay et al 2013 GLOBOCAN)

Five-year survival is highly dependent on the stage at diagnosis. Approximately one-fourth of CRC patients present metastatic disease at the time of diagnosis, and a further one-third will develop metastases during the course of the disease.

The introduction of oxaliplatin and irinotecan and the development of combination chemotherapy regimens FOLFOX (5-FU, folinic acid and oxaliplatin) (De Gramont et al 2000), FOLFIRI (5-FU, folinic acid, and irinotecan) (Douillard et al 2000) and FOLFOXIRI (5-FU, folinic acid, irinotecan and oxaliplatin) (Falcone et al 2007) has dramatically improved outcomes of patients with metastatic CRC (mCRC). Furthermore, recently, Trifluridine-Tipiracil, an oral cytotoxic agent, on the basis of RECOURSE trial, has been approved for the treatment of patients with refractory mCRC (Mayer et al 2015).

The recent advances made in the knowledge of tumor biology and molecular genetics have further influenced the treatment of mCRC patients, with the introduction of novel molecularly targeted agents: monoclonal antibodies (MoAbs) against vascular endothelial growth factor

(VEGF), bevacizumab (Hurwitz et al 2004); MoAbs against epidermal growth factor receptor (EGFR), cetuximab (Van Cutsem et al 2009) or panitumumab (Douillard et al 2010); the anti-angiogenic fusion decoy receptor blocking the activity of VEGFA, VEGFB, and placental growth factor, aflibercept (Van Cutsem et al 2012); and the multikinase receptor inhibitor regorafenib (Grothey et al 2013). Following the development of new treatment strategies and the increasing integration with surgery and local therapies, the outcome of patients with mCRC has progressively achieved a median survival of more than 30 months (Van Cutsem et al 2016). However, despite the recent developments, the efficacy of current treatment strategies, in particular for molecular targeted agents, is limited by the occurrence of drug-resistance mechanisms.

Whereas for bevacizumab, as well for other anti- angiogenic drugs, there is no predictive biomarker, it has been demonstrated that the efficacy of anti-EGFR monoclonal antibodies is limited to molecularly selected patients whose tumor is *RAS* and *BRAF* wild type. Several studies have provided new insights into molecular basis of EGFR inhibitors resistance and have identified mutations in *KRAS*, *NRAS*, *BRAF* and EGFR extracellular domain (ECD) as well as the amplification of *ERBB2* and *MET*, as biomarkers of both primary and/or acquired resistance (Sforza et al 2016, Schirripa et al 2016).

1.2 STUDY RATIONALE

Advances in the molecular understanding of the complex mechanisms of resistance to anti-EGFR MoAb, with the use of innovative technologies, such as liquid biopsies, are leading to the development of novel approach to revert or overcome resistance.

Rechallenge with an alternative anti-EGFR monoclonal antibody (MoAb) after failure with an agent of the same family has been proposed as strategy to overcome drug resistance. Recent studies have suggested a limited benefit from a retreatment strategy with anti-EGFR therapy in mCRC.

In a phase II study 39 patients with KRAS exon 2-wt mCRC who had previously progressed following an initial clinical benefit to cetuximab-based therapy, were retreated with cetuximab and irinotecan. Results demonstrated an overall response rate of 53.8 %, stable disease rate was 35.9 %, and the median progression-free survival was 6.6 months (Santini et al 2012). In PANERB trial 32 patients with KRAS wild-type mCRC were prospectively treated with cetuximab and irinotecan followed by panitumumab monotherapy after progression. In 11 patients who had previously responded to cetuximab and irinotecan, an objective response rate of 22 % to panitumumab, including a disease control rate (objective response plus stable

disease) of 73 % was observed (Wadlow *et al* 2012). In heavily pre-treated patients without acquired resistance to prior cetuximab-based regimens, panitumumab obtained 67 % disease control rate and 30 % objective response rate, with median PFS of 4.2 and median OS of 9.6 months (Pietrantonio et al 2013).

Trifluridine-Tipiracil, is an oral agent that combines trifluridine (TFD) and tipiracil hydrochloride (TP), that have provided benefit of 2 months in overall survival and have recently been approved for the treatment of refractory mCRC (Mayer et al 2015). Trifluridine-Tipiracil antitumor efficacy is mainly explained by the inhibition of thymidylate synthase (Santi et al 1971; Temmink et al., 2004), and the incorporation of FTD-TP into DNA with subsequent DNA damage (Tanaka et al., 2014; Suzuki et al, 2011; Matsuoka et al, 2015). Importantly, Trifluridine-Tipiracil exhibits antitumor activity against FU-resistant cell lines in preclinical xenograft models (Emura et al., 2004; Emura et al., 2004; van der Velden et al., 2016).

Panitumumab in combination with Trifluridine-Tipiracil have shown preliminary activity in KRAS WT tumor xenografts model (Tsukihara et al. 2015). Moreover, Baba et al have suggested that panitumumab interacts with TFD by targeting EGFR-mediated adaptive responses, enhancing anti-tumour effects when used in combination with Trifluridine-Tipiracil (Baba et al 2017).

The Phase I-II study (APOLLON) has recently evaluated panitumumab in combination with Trifluridine-Tipiracil in patients with *RAS* WT mCRC, refractory to standard chemotherapies (NCT02613221). The recommended phase II dose of this combination was reported as standard dose (panitumumab: 6 mg/kg on days 1 and 15, every 4 weeks; Trifluridine-Tipiracil: 35 mg/m2 BID on days 1-5 and 8-12, every 4 weeks) (Kato et al 2017).

Preliminary data have shown favorable antitumor activity with an acceptable safety profile: median PFS, RR and DCR were 5.8 months (95% CI: 4.5–6.5), 37.0% and 81.4%, respectively (n = 54); The most common grade (G) ≥3 treatment-emergent adverse events (n = 55) were neutropenia (G3: 30.9%, G4: 16.4%), febrile neutropenia (G3: 10.9%), stomatitis (G3: 9.1%), dermatitis acneiform (G3: 9.1%), fatigue (G3: 3.6%) and hypomagnesemia (G3: 3.6%), no treatment-related deaths or unexpected safety signals was reported.

2. AIMS OF THE STUDY

2.1 Study Hypothesis

The aim of this study is to investigate a rechallenge strategy of panitumumab in combination with Trifluridine-Tipiracil as compared to standard third line therapy (Trifluridine-Tipiracil) in

patients with *RAS* WT mCRC, treated in first line with chemotherapy in combination with an anti-EGFR drug and have obtained a clinical benefit (complete or partial response) from treatment.

2.2 Objectives of the study

2.2.1 Primary Objective

- To explore the efficacy of panitumumab in combination with Trifluridine-Tipiracil vs standard third line therapy (Trifluridine-Tipiracil) as measured by PFS

2.2.2Secondary Objectives

- Compare the activity of panitumumab in combination with Trifluridine-Tipiracil vs standard third line therapy (Trifluridine-Tipiracil) as measured by ORR
- Compare the efficacy of panitumumab in combination with Trifluridine-Tipiracil vs standard third line therapy (Trifluridine-Tipiracil) as measured by OS
- To explorethe safety and tolerability of panitumumab in combination with Trifluridine-Tipiracil vs standard third line therapy (Trifluridine-Tipiracil)

2.2.3 Exploratory secondary objectives

- To explore the mutational profile and evaluate acquired mutations in plasma circulating cell-free DNA (cfDNA) and to explore the relationship with panitumumab in combination with Trifluridine-Tipiracil activity

2.3 Endpoints of the study

2.3.1 Primary Endpoint

- PFS: defined as the time from randomization to the earliest documented disease progression or death due to any cause of panitumumab in combination with Trifluridine-Tipiracil vs Trifluridine-Tipiracil

2.3.2 Secondary Endpoints

- ORR: per the Response Evaluation Criteria in Solid Tumors (RECIST), version 1.1 (v1.1), defined as the number of patients achieving an overall best response of complete or partial response divided by the total number of patients
- OS: defined as the time from randomization to death due to any cause of panitumumab in combination with Trifluridine-Tipiracil vs Trifluridine-Tipiracil
- Safety analysis: defined as the evaluation of incidence and severity of Adverse Events (AEs), graded according to National Cancer Institute (NCI) Common Terminology Criteria for Adverse Events (CTCAE), version 5.0 (v. 5.0) of panitumumab in combination with Trifluridine-Tipiracil vs standard third line therapy (Trifluridine-Tipiracil)

2.3.3 Exploratory secondary endpoints

- Genomic analysis of blood samples collected baseline, during treatment period and at end of treatment

3. SELECTION OF PATIENTS

3.1 Inclusion Criteria

Patients must meet the following criteria for study entry:

- Signed informed consent to any study-specific procedure.
- Age ≥18 years
- Eastern Cooperative Oncology Group (ECOG) performance status of 0 or 1 (see Appendix 1)
- Histologically confirmed adenocarcinoma of the colon or rectum
- Stage IV American Joint Committee on Cancer [AJCC] 8th edition
- Measurable tumour lesions according to RECIST v1.1 (with at least one measurable lesion)
- Life expectancy of at least 3 months
- *RAS* (NRAS and KRAS exon 2,3 and 4) wild-type in tissue at initial diagnosis
- Experienced disease progression on two prior systemic chemotherapy regimens for mCRC, as follows:

1. Received a first line therapy containing an anti-EGFR agent (panitumumab or cetuximab) and obtained an objective response (complete or partial response) from treatment;

2. Progressed to a second line regimen of standard chemotherapy (including fluoropyrimidines, irinotecan, oxaliplatin, bevacizumab or aflibercept)

Note:

- Patient who is refractory to chemotherapy is defined as a patient who didn't respond (obtaining a PR or CR) from the beginning of the treatment (primary refractory) or, after initial response, experiences progression while on treatment (secondary refractory);
- Patient who is intolerant to chemotherapy is defined as a patient who has been withdrawn from standard treatment due to unacceptable toxicity warranting discontinuation of treatment and precluding retreatment with the same agent prior to progression of disease. This patient will also be eligible to enter the study;

- Able to take medications orally (*i.e*., no feeding tube)
- Available and adequate baseline tumour tissue sample
- Adequate organ function as defined by the following laboratory values:

- Hemoglobin value of $\geq$9.0 g/dL.
- Absolute neutrophil count of $\geq 1.5\times 10^9$/L by International Units (IU).
- Platelet count $\geq 100 \times 10^9$/L
- Total serum bilirubin of $\leq$1.5 mg/dL (except for Grade 1 hyperbilirubinemia due solely to a medical diagnosis of Gilbert's syndrome)
- Aspartate aminotransferase (AST) and alanine aminotransferase (ALT) $\leq 3.0\times$upper limit of normal (ULN); if liver function abnormalities are due to underlying liver metastasis, AST and ALT $\leq 5 \times$ ULN
- Creatinine clearance $\geq$ 60 mL/min (assessed with Cockcroft-Gault formula)

- Women of childbearing potential must have been tested negative in a serum pregnancy test within 14 days prior to first day of test drug administration

  Note:

- Female participants of childbearing potential and male participants with partners of childbearing potential must agree to use a highly effective method of birth control (*i.e*., pregnancy rate of less than 1% per year) during the study and for 6 months after the discontinuation of study medication

- A women is not considered to be of childbearing potential if she has become amenorrheic for > 12 months and has follicle-stimulating hormone level ≥40 IU/L

Contraceptive methods that result in a low failure rate when used consistently and correctly include methods such as combined hormonal contraception associated with inhibition of ovulation (oral, intravaginal, transdermal), progestogen-only hormonal contraception associated with inhibition of ovulation (oral, injectable, implantable), some intrauterine devices (IUDs), intrauterine hormone-releasing system (IUS), true sexual abstinence, bilateral tubal occlusion, or a female partner who is not of childbearing potential or a male partner who has had a vasectomy. Women and female partners using hormonal contraceptive must also use a barrier method *i.e.* condom or occlusive cap (diaphragm or cervical/vault caps).

3.2 Exclusion Criteria

A subject is excluded from the study if any of the following criteria is met:

- Unlikely to cooperate in the study
- Pregnancy, breastfeeding or possibility of becoming pregnant during the study
- Hypersensitivity to the active substances or to any of the excipients of Trifluridine-Tipiracil or panitumumab
- More than two previous lines of treatment
- Previously received Trifluridine-Tipiracil
- Other concurrently active malignancies, excluding skin basal cell and cervix in situ cancers cured by adequate treatment
- Active or untreated Central Nervous System (CNS) metastases are excluded. Patients with treated and asymptomatic CNS metastases are eligible if they meet all of the following:
- Evaluable disease outside the CNS
- No evidence of intracranial haemorrhage or spinal cord haemorrhage
- Corticosteroids ant anti-convulsivant at a stable dose
- Myocardial infarction within the last 12 months, severe/unstable angina, symptomatic congestive heart failure New York Heart Association (NYHA) class III or IV

- Uncontrolled tumor-related pain. Patients requiring pain medications must be on a stable regimen at study entry
- Symptomatic lesions (e.g. bone metastases) amenable to palliative radiotherapy should be treated prior to start of study treatment
- Active infection (e.g., body temperature ≥38°C due to infection)
- Pulmonary fibrosis or cerebrovascular disorder at the time of screening
- Uncontrolled diabetes
- Active gastrointestinal haemorrhage or intestinal obstruction at the time of screening
- In the investigator's opinion, significant malabsorption syndrome, significant chronic digestive or gastrointestinal inflammatory syndrome
- Known human immunodeficiency virus (HIV) or acquired immunodeficiency syndrome (AIDS)-related illness, or active hepatitis B or C.
- Autoimmune disorders or history of organ transplantation who require immunosuppressive therapy
- Psychiatric disease that may increase the risk associated with participation or drug administration, or may interfere with the interpretation of results
- Rare hereditary problems of galactose intolerance, the Lapp lactase deficiency, or glucose-galactose malabsorption
- Major surgery within 4 weeks prior to first day of study drug administration
  Note: indwelling catheter and Portacath® implantation are allowed
- Any anticancer therapy within 3 weeks prior to first day of study drug administration
- Radiation therapy within 3 weeks prior to first day of study drug administration
- Unresolved toxicity of ≥Grade 2 CTCAE attributed to any prior therapy (excluding anaemia, alopecia, skin pigmentation, and platinum-induced neurotoxicity)

4. STUDY DESIGN AND PROCEDURES

This is a randomized phase II, open label, two arm study, evaluating the efficacy of panitumumab in combination with Trifluridine-Tipiracilas third line therapy, after a first line containing an anti-EGFR agent panitumumab(at least 70% of study population) or cetuximab. The primary objective is to evaluate the efficacy of panitumumab in combination with Trifluridine-Tipiracil (as measured by PFS) in pretreated mCRC patients. The secondary objective is to evaluate the ORR, OS and safety. Patients will be randomized in a 1:1 ratio to receive:

Arm A: Trifluridine-Tipiracil

Arm B: Panitumumab + Trifluridine-Tipiracil

4.1 Sample size

A total of 112 patients, 56 for arm, will be enrolled.

4.2 Study Duration

It is planned that patient's enrolment will be completed in 18 months with an expected total study duration of approximately 30months considering an additional 12 months of follow up from last patient enrolled.

4.3 Treatment plan

Eligible Patients (see inclusion and exclusion criteria see sections 3.1 and 3.2) will be randomized to receive a third line treatment with panitumumab in combination with Trifluridine-Tipiracil or Trifluridine-Tipiracil as monotherapy (see Study Design, Figure 1).

Randomization will be stratified by baseline Eastern Cooperative Oncology Group (ECOG PS: 0 vs 1), prior anti-EGFR agent administered (panitumumab at least 70% of study population vs cetuximab), and tumor sidedness (right vs left colon). In particular, the tumors located from the caecum to a point approximately half the two-thirds of the way along the transverse colon will be considered as "right colon", whilst the tumors located from the distal third of the transverse colon to the rectum will be considered "left colon".

Treatment will be administered in 28-days cycles until disease progression, unacceptable toxicity, withdrawal of consent or death due to any cause.

All patients will be closely monitored for safety and tolerability during all cycles of therapy, at the treatment discontinuation visit, and during the follow-up period. The NCI v.5.0 will be used to characterize the toxicity profile of the study treatments on all patients.

Patients who discontinue treatment for reasons other than disease progression (e.g., toxicity) will continue scheduled tumor assessments until disease progression, withdrawal of consent,

study termination by Sponsor, or death, whichever occurs first. In the absence of disease progression, tumor assessments should continue regardless of whether patients start a new anti-cancer therapy, unless consent is withdrawn. All patients will be followed for survival unless consent is withdrawn (see section 4.4.5).

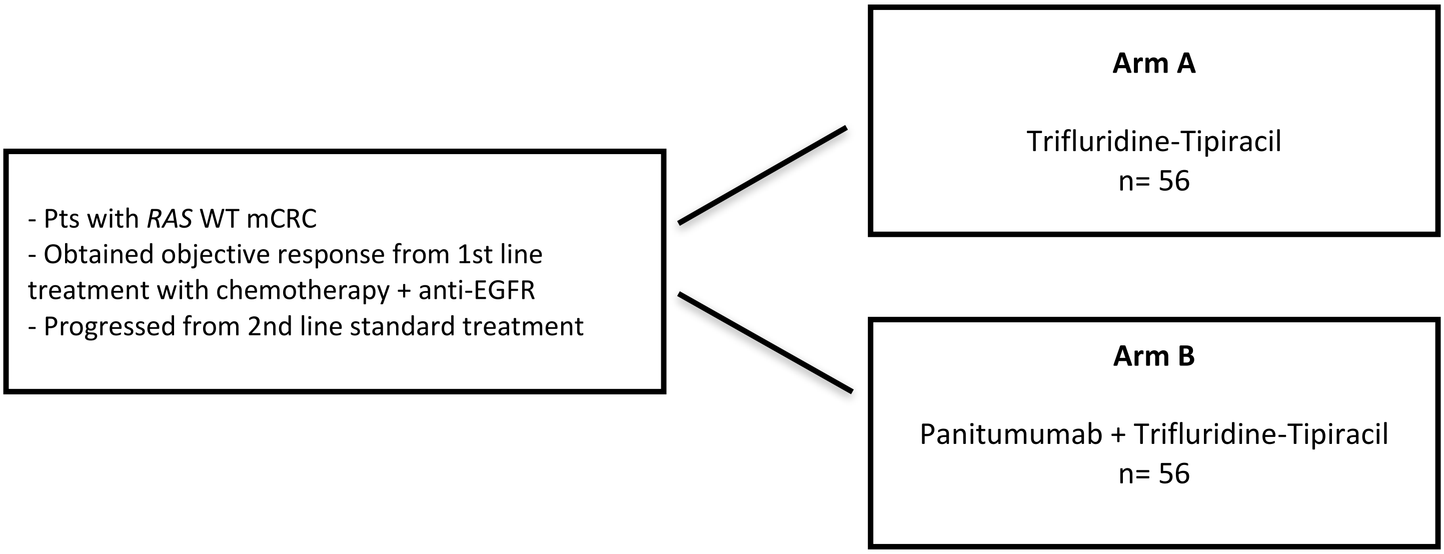

Figure 1. Study Design

4.3.1 Treatment dosage and schedule

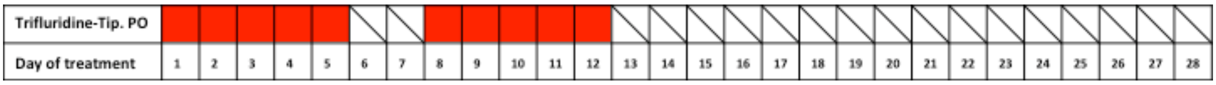

Figure 2.1. Treatment Schedule: Arm A (Trifluridine-Tipiracil)

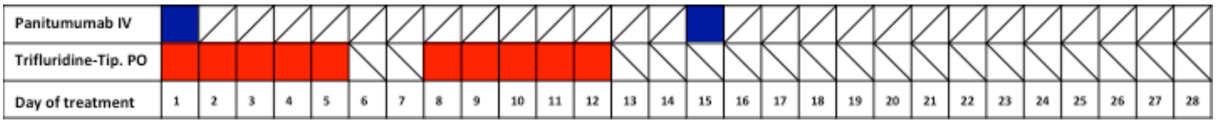

Figure 2.2. Treatment Schedule: Arm B (Panitumumab + Trifluridine-Tipiracil)

Panitumumab administration

Panitumumab will be administered as a 6 mg/kg intravenous infusion over 60 minutes every 2 weeks (q2w) of a 28-day cycle (Day 1 and Day 15) (see Figure 2.2)

Note: If the subject's weight changes by ≥ 10% during the course of the study, the dose of panitumumab will be recalculated. No ideal body weight should be used for the calculation of BSA.

Panitumumab must be diluted in pyrogen-free 0.9% sodium chloride for injection using aseptic technique to a recommended total volume of 100 mL. Doses higher than 1000 mg should be diluted to 150 mL. The maximum concentration of the final solution to be infused should not exceed 10 mg/mL.

The diluted products should be used within 6 hours of preparation if stored at room temperature, or within 24 hours of dilution if stored refrigerated at 2ºC and 8ºC. Recommended safety measures for preparation and handling of drugs include laboratory coats and gloves.

For details on prepared drug storage and use time of drugs under room temperature and refrigeration, please refer to pharmacy reference sheets.

Pre-emptive skin treatment regimen is mandatory, if there are not contraindications and it must be administered one day before the administration of the first Panitumumab dose and continued through weeks 1 to 6 (Please refer to paragraph 4.3.3 "Concomitant medication" and Permissible concomitant medications and treatments" for more details).

Trifluridine-Tipiracil administration

Trifluridine-Tipiracil will be administered at 35 mg per square meter orally twice daily, with a glass of water within 1 hour after completion of morning and evening meals, 5 days a week, with 2 days of rest, for 2 weeks, followed by a 14-day rest period (see Figure 2.1 and 2.2). One treatment cycle with Trifluridine-Tipiracil consists of the following:

Days 1-5: Trifluridine / Tipiracil (35 mg/m^2/dose) orally twice daily

Days 6-7: Rest

Days 8-12: Trifluridine / Tipiracil(35mg/m^2/dose) orally twice daily

Days 13-28: Rest

Note:

- If at the beginning of the next treatment cycle, a patient's body weight decreases by ≥10% from baseline, the body surface area (BSA) and the dosage of Trifluridine-Tipiracil has to be adjusted (See Table 1 and Table 8).
- No increase in Trifluridine-Tipiracil dose due to increase in BSA is permitted.
- If doses are missed or held, the patient should not make up for missed doses.
- Any missed doses reported by the patient should be recorded in the patient's diary.
- Extension of study treatment into Days 6 to 7 or into the rest period (Days 13 through 28) is not permitted.

For information on the formulation, packaging and handling of Trifluridine-Tipiracil, see Trifluridine-Tipiracil, Lonsurf ® prescribing information.

**Trifluridine-Tipiracil dose (2 x daily)**	**BSA (m^2)**	**Dosage in mg (2x daily)**	**Total daily dose (mg)**	**Tablets per dose**	
				**15 mg**	**20 mg**
**35 mg/m^2**	<1.07	35	70	1	1
	1.07 – 1.22	40	80	0	2
	1.23 – 1.37	45	90	3	0
	1.38 – 1.52	50	100	2	1
	1.53 – 1.68	55	110	1	2
	1.69 – 1.83	60	120	0	3
	1.84 – 1.98	65	130	3	1
	1.99 – 2.14	70	140	2	2
	2.15 – 2.29	75	150	1	3
	≥ 2.30	80	160	0	4

Table 1. Number of tablets of Trifluridine- Tipiracil per dose

4.3.2 Dose Modifications, delays ad interruptions criteria

General Recommendation

Subjects should be assessed for toxicity before each treatment cycle. Toxicity will be graded according to the NCI CTCAE Version 5.0. Reasons for dose modifications, delays or interruptions, the supportive measures taken, and the outcome, will be documented in the patient's chart and recorded in the eCRF.

<u>Guidelines to be followed in the case of AEare :</u>

- For toxicities considered by the investigator unlikely to develop into serious or life-threatening events (e.g. alopecia, altered taste etc.), treatment will be continued at the same dose without reduction or interruption.
- Where several toxicities with different grades or severity occur at the same time, the dose modifications applied should be the greatest reduction applicable.
- Once a dose has been reduced it should not be increased at a later time.
- If toxicity requires a dosing delay of all study drugs for more than four weeks, the patient will be withdrawn from the study for toxicity reasons.

Note:

For patients randomized to Arm B (Panitumumab+ Trifluridine-Tipiracil) (Figure 2.2):

– If, in the opinion of the Investigator, a toxicity is considered to be due solely to one drug, the dose of the other drug does not require modification.
– If a delay related to a treatment alone is required, the other study drug should be delayed as well.

Grade of Event	Management/Dose regulation for study drug
**Grade 1**	no change in dose
**Grade 2**	Tolerable: no change in dose<br>Non tolerable: hold until ≤ grade 1 – resume at same dose
**Grade 3**	Hold* until ≤ grade 2 – resume at 1 dose level lower if indicated
**Grade 4**	Hold* until ≤ grade 2 – resume at 1 dose level lower
* Patients requiring a delay of > 4 weeks should discontinue protocol therapy	

Table 2. Study drug dose modifications according to toxicity grade

Dose delay and modification criteria for panitumumab-related adverse events

In the event of panitumumab-related adverse events patients should follow the dose interruption, resumption and reduction criteria stated in Table 2.

Note: dose modifications levels for panitumumab are stated in Table 3.

**Starting dose**	100% of original dose
**Level 1 Dose reduction**	80% of original dose
**Level 2 Dose reduction**	60% of original dose

Table 3. Panitumumab dose modification levels

It is allowed, according to the Investigator's opinion, with holding panitumumab administration, up to a maximum of four weeks, for the following reasons:

- Symptomatic skin- or nail-related toxicity requiring narcotics, systemic steroids, or felt to be intolerable by the subject (see Table 4)
- Skin or nail infection requiring IV antibiotic or IV antifungal treatment
- Any skin- or nail-related serious adverse event
- Symptomatic hypomagnesaemia and/or hypocalcaemia that persists despite magnesium and/or calcium replacement (see Table 5)

Note: for detailed toxicities grading refer to NCI CTCAE v. 5.0

**Occurrence of skin toxicity(s):≥ grade 3**	**Administration of Panitumumab**	**Outcome**	**Dose regulation**
Initial occurrence	Withhold 1 or 2 doses	Improved (< grade 3)	Continuing infusion at 100% of original dose
		Not recovered	Discontinue
At the second occurrence	Withhold 1 or 2 doses	Improved (< grade 3)	Continuing infusion at 80% of original dose
		Not recovered	Discontinue
At the third occurrence	Withhold 1 or 2 doses	Improved (< grade 3)	Continuing infusion at 60% of original dose
		Not recovered	Discontinue
At the fourth occurrence	Discontinue	-	-

Table 4: Panitumumab dose modifications and delay according to skin toxicities

**Event**	**Grade**	**Adjustment**
Symptomatic hypomagnesemia *First occurrence*	any	Hold Panitumumab until resolution and restart at 100% dose level with Mg++ supplementation
Symptomatic hymomagnesemia in patients treated at 100% or 80% dose level *Recurring*	any	Restart Panitumumab at 80% dose level or 60% dose level respectively

Diarrhea<br>*First Occurrence*	3 or 4	Hold Panitumumab until resolution and restart at 100% dose level
Diarrhea in patients treated at 100% or 80% dose level<br>*Recurring*	3 or 4	Restart Panitumumab at 80% dose level or 60% dose level respectively
Any haematologic or non-haematologic toxicity	4	Hold Panitumumab until resolution

Table 5: Panitumumab dose modifications and delay according to other toxicities

Dose delay and modification criteria for Trifluridine-Tipiracil related adverse events

In the event of haematological and/or non-haematological toxicities patients should follow the dose interruption, resumption and reduction criteria stated in Table 6, Table 7, Table 8.

- If the toxicities that require trifluridine / tipiracil dose reductions recur after dose reduction to 20 mg/m², trifluridine / tipiracil should be discontinued.
- A maximum of three dose reduction(s) is permitted to a minimum dose of 20 mg/m² (total 40 mg/m²/day) in 5 mg/m² steps. Dose re-escalation is not permitted at any time.

**Withhold Trifluridine-Tipiracil if any of these AE occurs:**	**Resumption criteria[a]**
Neutrophils<0.5 x 10^9/L	Neutrophils≥1.5 x 10^9/L;
Febrile Neutropenia	Febrile neutropenia resolved
Platelets<50 x 10^9/L	Platelets≥75 x 10^9/L
Grade 3 or 4 non hematologicAEs	Grade ≤1 non hematologicAEs

[a]Resumption criteria applied to the start of the next cycle for all patients regardless of whether or not the interruption criteria were met

Table 6 - Dose interruption and resumption criteria for Trifluridine-Tipiracil related AEs

**Adverse events that require dose modifications:**	**Recommended dose modifications:**
· Febrile neutropenia<br>· Grade 4 neutropenia (<0.5 x 10^9/L) or thrombocytopenia (<25 x 10^9/L) that results in more than 1 week delay in start of next cycle<br>· Non-hematologic Grade 3 or Grade 4 adverse reaction (except for Grade 3 nausea and/or vomiting controlled by antiemetic therapy or diarrhea responsive to antidiarrheal medicinal products)	· Interrupt dosing until toxicity resolves Grade ≤1 .<br>· When resuming dosing, decrease the dose level by 5 mg/m²/dose from the previous dose level (Table 8: dose reduction).<br>· Dose reductions are permitted to aminimum dose of 20 mg/m²/dose twice daily.<br>· Do not increase dose after it has been reduced.

Table 7– Recommended dose modifications for Trifluridine/Tipiracil in case of hematological and non-hematological adverse reactions

**Trifluridine / Tipiracil dose (2x daily)**	**BSA (m^2)**	**Dosage in mg (2x daily)**	**Total daily dose (mg)**	**Tablets per dose**	
				**15 mg**	**20 mg**
**Level 1 Dose reduction: From 35 mg/m^2 to 30 mg/m^2**					
	**<1.09**	**30**	**60**	**2**	**0**
	**1.09 – 1.24**	**35**	**70**	**1**	**1**
	**1.25 – 1.39**	**40**	**80**	**0**	**2**
	**1.40 – 1.54**	**45**	**90**	**3**	**0**
**30 mg/m^2**	**1.55 – 1.69**	**50**	**100**	**2**	**1**
	**1.70 – 1.94**	**55**	**110**	**1**	**2**
	**1.95 – 2.09**	**60**	**120**	**0**	**3**
	**2.10 – 2.28**	**65**	**130**	**3**	**1**
	**≥ 2.29**	**70**	**140**	**2**	**2**
**Level 2 Dose reduction: From 30mg/m^2 to 25mg/m^2**					
	**<1.10**	**25**	**50**	**2 (PM)[a]**	**1 (AM)[a]**
	**1.10 – 1.29**	**30**	**60**	**2**	**0**
	**1.30 – 1.49**	**35**	**70**	**1**	**1**
**25 mg/m^2**	**1.50 – 1.69**	**40**	**80**	**0**	**2**
	**1.70 – 1.89**	**45**	**90**	**3**	**0**
	**1.90 – 2.09**	**50**	**100**	**2**	**1**
	**2.10 – 2.29**	**55**	**110**	**1**	**2**
	**≥2.30**	**60**	**120**	**0**	**3**
**Level 3 Dose reduction: From 25 mg/m^2 to 20 mg/m^2**					
	**<1.14**	**20**	**40**	**0**	**1**
	**1.14 – 1.34**	**25[a]**	**50[a]**	**2(PM)[a]**	**1(AM)[a]**
	**1.35 – 1.59**	**30**	**60**	**2**	**0**
**20 mg/m^2**	**1.60 – 1.94**	**35**	**70**	**1**	**1**
	**1.95 – 2.09**	**40**	**80**	**0**	**2**
	**2.10 – 2.34**	**45**	**90**	**3**	**0**
	**≥2.35**	**50**	**100**	**2**	**1**

[a]At a total daily dose of 50 mg, patient should take 1 x 20mg tablet in the morning and 2 x 15mg tablets in the evening.

Table 8 - Trifluridine/Tipiracil dose reduction levels and number of tablets per dose

Criteria to resume treatment

Subjects may resume treatment with study drug when the drug-related AE(s) resolve to Grade ≤ 2 or baseline value, as per Table 2, with the exception of Trifluridine-Tipiracil toxicities, as per Table 6, Table 7, Table8.

If the criteria to resume treatment are met, the subject should restart treatment at the next scheduled time-point per protocol.

If treatment is delayed >4 weeks, the subject must be permanently discontinued from study. However, the patient can continue any treatment as per local standard practice and must be followed up with the study procedures.

Discontinuation criteria

Treatment with panitumumab should be permanently discontinued for the following reasons:

- Progressive disease during treatment
- Patient's refusal
- Interstitial lung disease (ILD)
- Grade 3 or 4 allergic/hypersensitivity reactions
- Grade 3 or 4 diarrhea not recovered withholding treatment for 1 or 2 doses
- Unacceptable AEs, or change in underlying condition such that the patient can no longer tolerate therapy
- Study drug dose reduction by more than two dose levels
- Investigator's decision
- If female, a pregnancy test result consistent with pregnancy
- Development of any intercurrent illness or situation that may, in the judgment of the investigator affect assessments of clinical status and study endpoints to a relevant degree.

Treatment with Trifluridine-Tipiracil should be permanently discontinued for the following reasons:

- Progressive disease during treatment
- Patient's refusal
- Unacceptable AEs, or change in underlying condition such that the patient can no longer tolerate therapy.
- Study drug dose reduction until < 20 mg/m²

- Investigator's decision
- If female, a pregnancy test result consistent with pregnancy
- Development of any intercurrent illness or situation that may, in the judgment of the investigator affect assessments of clinical status and study endpoints to a relevant degree.

Patients have the right to withdraw from the treatment or from the study at any time and irrespective of the reason. When applicable, patients should be informed of circumstances under which their participation may be terminated by the Investigator without their consent. The Investigator may withdraw patients from the study in the event of intercurrent illness, AEs, treatment failure after a prescribed procedure, lack of compliance with the study and/or study procedures (e.g., dosing instructions, study visits), or any reason where it is felt by the Investigator that it is in the best interest of the patient to be terminated from the *study*. Any administrative or other reasons for withdrawal must be documented and explained to the patient.

Any subject who definitively interrupts study treatment will be evaluated at the End-of-treatment Visit conducted 21 days (±7 days) after permanently stopping study treatment (see section 4.4.4).

In all cases, the reason for definitive interruption of study treatment mustbe entered in the electronic Case Report Form (eCRF) and in the subject's medical records. Patients who withdraw from the study will not be replaced. Only patients included in the study that have not started treatment with the first dose of drugs will be replaced.

4.3.3Concomitant Treatments

The patient must notify the investigational site about any new medications he/she takes after the start of the study drug. All medications (other than study drug) and significant non-drug therapies (including physical therapy, herbal/natural medications and blood transfusions) administered during the study must be listed on the Concomitant Medications or the Surgical and Medical Procedures eCRF.

Patients taking concomitant medications chronically should maintain the same dose and dose schedule throughout the study if medically feasible. However, if a concomitant medication is used intermittently during the study, this medication should be avoided on these days, if medically feasible.

Permissible concomitant medications and treatments

- Standard therapies for concurrent medical conditions. Prophylactic anti-emetics may be administered according to standard practice
- G-CSF and other haematopoietic growth factors may be used during the study for the management of acute toxicity such as febrile neutropenia when clinically indicated or at the discretion of the investigator; however, they may not be substituted for a required dose reduction. The use of granulocyte colony stimulating factors (G-CSF) as secondary prophylaxis is allowed, as per standard treatment (J. Klastersky et al 2016, ESMO guidelines). Long-term administration of erythropoietin is permitted
- Bisphosphonates and denosumab
- Subjects taking narrow therapeutic index medications, such as warfarin, quinidine, cyclosporine, and digoxin, should be monitored proactively. A subject treated with warfarin or heparin will be allowed to participate provided that the anticoagulant dose and PT-INR and a-PTT values are stable. Close monitoring (evaluation - once weekly) is recommended. If either value is above the therapeutic range, the anti-coagulant dose should be modified and the assessments should be repeated once weekly until the value is stable.
- Palliative radiotherapy may be given for control of pain or for other reasons without curative intent.
- Pre-emptive skin treatment regimen is mandatory, if there are not contraindications and it must be administered one day before the administration of the first panitumumab dose and continued through weeks 1 to 6. It consists of:

  -skin moisturizer applied to face, hands, feet, neck, back, and chest daily in the morning on rising;

  - sunscreen (PABA free, SPF ≥ 15, UVA and UVB protection) applied to exposed skin areas before going outdoors;

  - topical steroid (1% hydrocortisone cream) applied to face, hands, feet, neck, back, and chest at bedtime

  - doxycycline 100 mg twice daily. During the assumption of doxycycline, the patient must be advised to drink plenty of liquids to reduce the risk of esophageal irritation and ulceration; he/she must take doxycycline in upright position and must not lie down for an hour after taking the drug. The patient should not take iron supplements, multivitamins, calcium supplements, antacids, or laxatives within 2 hours before or after taking

doxycycline. Please remember that the absorption of tetracyclines is reduced when taken with foods, especially those containing calcium.

Prohibited concomitant medications and treatments

- No other systemic anti-cancer therapy
- Caution is required when using medicinal products that are human thymidine kinase substrates, e.g., zidovudine. Such medicinal products, if used concomitantly with Trifluridine-Tipiracil, may compete with the effector, Trifluridine, for activation via thymidine kinases. Therefore, when using antiviral medicinal products that are human thymidine kinase substrates, monitor for possible decreased efficacy of the antiviral medicinal product, and consider switching to an alternative antiviral medicinal product that is not a human thymidine kinase substrate, such as lamivudine, zalcitabine, didanosine and abacavir.

4.4 Schedule of Assessments and Procedures

All screening/baseline assessments must be performed as outlined in schedule assessment flow chart (see Tables 9)

Eligibility for the study will be determined by the Investigator, from the mandatory screening/baseline assessments, performed during screening and according to the study inclusion/exclusion criteria.

First dosing of study drugs will be determined by the patient's eligibility and the laboratory assessments done prior to dosing.

Patients will be assessed for AEs at each clinical visit according to NCI CTC-AE v. 5.0 criteria and as necessary throughout the study.

Patients who discontinue study drug for any reason (e.g., AEs, etc.) other than disease progression will continue to be followed.

Progression of disease will be documented, as well as the initiation of another cancer therapy

4.4.1 Procedures for Enrolment of Eligible Patients

A patient who has fulfilled the entry criteria will be randomized and given an identifying number. Each identifying number will be unique to the patient for whom it is issued. A patient number will not be re-used if the patient leaves the study. Under no circumstances will patients

who enroll in this study and have completed treatment as specified be permitted to re-enroll in the study. A Patient Enrolment and Identification Code List must be maintained by the Investigator.

4.4.2 Screening and Baseline Assessments

All screening/baseline evaluations must be performed within 28 days prior to randomization. Patients who fulfill all the inclusion and none of the exclusion criteria will be accepted into the study. The following clinical assessments and procedures must be completed for all patients enrolled in this study at screening/baseline and during study visits. Screening procedures could be completed during multiple visits but must be completed within the specified timeframes.

Screening procedures to be completed or that must be available within 28 days prior to randomization

- Informed Consent Form. No screening procedures may be performed until written informed consent has been obtained. Informed Consent Forms for enrolled patients and for patients who are not subsequently enrolled will be maintained at the study site
- Histologically confirmed diagnosis of colorectal adenocarcinoma, *RAS* status.
- Medical history (including allergies, relevant medical history, previous and current diseases, all ongoing medications) and Demographic data (age, gender, race);
- Tumor assessment: CT or MRI eligibility scan (chest, abdomen, pelvis, and other suspected sites as applicable) meeting the standard of care for the imaging of the respective organ system(s). All measurable disease must be documented.
  Note: Tumor assessment performed prior to patient ICF signature may be used if the date of evaluation is within 28 days prior to randomization.
- 12-lead ECG
- ECOG Performance status
- Physical exam including height (screening only) and weight and skin examination. Changes from baseline abnormalities should be recorded at each subsequent physical examination. New or worsened abnormalities should be recorded as AEs if appropriate
- Vital signs (respiratory rate, pulse, blood pressure and temperature) will be obtained in the same position
- Haematology (including: haemoglobin, haematocrit, Platelet Count, white blood cell [WBC] count, absolute neutrophil count [ANC]); All laboratory tests performed during

patient's visit and inter visits laboratory tests (unscheduled tests) could be assayed in any certified laboratory (upon communication to the Sponsor of laboratory normal ranges).

- All samplings for biochemistry should be taken in fasting conditions.
- Biochemistry (including: glucose, blood urea nitrogen (BUN), creatinine or creatinine clearance, sodium, potassium, calcium, magnesium, chloride, total bilirubin with fractionation into direct and indirect (if total bilirubin elevated), alkaline phosphatase, aspartate aminotransferase [AST, or serum glutamic oxalo-acetic transaminase, SGOT], alanine aminotransferase [ALT, or serum glutamic-pyruvic transaminase, SGPT]) and lactate dehydrogenase (LDH); serum B-HCG test for women potentially fertile
  Note: serum B-HCG test should be obtained within 14 days prior to C1D1
- Urine samples for qualitative (dipstick) analysis, (protein, glucose, urobilinogen, RBC, and WBC)
- Basal tumor markers: CEA and CA19.9
- AEs (including SAEs) from time signed Informed Consent is obtained until first dose of study drugs
- Peripheral blood samples for plasma biomarker analyses

Treatment assignment

After written informed consent has been obtained and eligibility established, each patient will be randomized as per Study Design (see Figure 1). Randomization should be performed within 3 days to C1D1.

4.4.3Assessment during treatment

Patients should receive study drug according to treatment schedule (Figure 2.1 and 2.2)

- ECOG performance status (Day 1 and day 15 of each cycle)
- Physical exam and Vital signs (as indicated above) at every visit, prior to any treatment cycle. (day 1 and day 15 of each cycle)
- Haematology (including haemoglobin, haematocrit, platelet count, WBC, ANC) within 72 hours prior to day 1 of each treatment cycle. (day 1 of each cycle)
- Biochemistry (including glucose, BUN, creatinine or creatinine clearance, sodium, potassium, calcium, magnesium, chloride, total bilirubin with fractionation into direct and indirect (if total bilirubin elevated), alkaline phosphatase, AST [SGOT], ALT [SGPT] and lactate dehydrogenase (LDH), prior to day 1 of each treatment cycle.

Note: procedures (haematology, biochemistry) have not to be repeated if performed as baseline (screening) within 7 days prior to randomization.

- Evaluation of tumor markers response: CEA and CA19.9 every 8 weeks ± 7 days.
- Evaluation of tumor response by CT, or MRI eligibility scan (chest, abdomen, pelvis, and other having resulted positive during baseline staging) conforming to RECIST v1.1 must be performed every 8 weeks ± 7 days until PD on treatment. Tumor assessments should be performed on this schedule regardless of whether study treatment has been administered or held, and also if the patient is off treatment due to reason other than PD (i.e. refusal or unacceptable toxicity).
- Peripheral blood samples for exploratory biomarker studies every 8 weeks ± 7 days, the first day before drugs administration of a treatment cycle until PD.
- AEs (including SAEs) throughout the study.

4.4.4 Assessments at End of Treatment

The End-of-Treatment assessments should be performed no later than 21 days+/- 7 days after patient's last dose of study medication. If the patient will be starting new anticancer therapy within the 21 days +/- 7 days after the last dose of study medication, the withdrawal visit should be performed prior to the start of new anticancer therapy.

- ECOG performance status
- Haematology (including haemoglobin, haematocrit, platelet count, WBC, ANC)
- Biochemistry (including glucose, BUN, creatinine or creatinine clearance, sodium, potassium, calcium, magnesium, chloride, total bilirubin with fractionation into direct and indirect (if total bilirubin elevated), alkaline phosphatase, AST [SGOT], ALT [SGPT] and lactate dehydrogenase (LDH)
- Concomitant disease and medications
- Documentation of AEs (CTCAE v 5.0 grading)
- Peripheral blood samples for plasma biomarker analyses

4.4.5 Follow-Up Assessments

All subjects who finish treatment, whichever the reason, will enter the follow-up. All subjects will be followed until death and data on subsequent treatment will be collected. Follow-up information on tumor status and vital status will be updated every 3 months until death. For patients with no progressive disease after terminating study treatment (i.e. treatment refusal,

unacceptable toxicity), tumor assessments, blood samples for plasma biomarker analyses, will be performed every 8 weeks ± 7 days until PD
If lost to follow-up, the Investigator should make every effort to contact the patient by telephone or by sending a registered letter to establish as completely as possible the reason for the withdrawal. A complete final evaluation at the time of the patient's withdrawal should be made with an explanation of why the patient is withdrawing from the study.

4.4.6 Collection of CT scans

It is required that CT scans or other relevant radiologic examinations used for staging and restaging procedures are collected and made available for eventual revision of response and time-to-progression definition.

Procedure	Screening period/Inclusion	On-treatment period			Withdrawal visit
		Cycles (28 days)		Based on Patient's status	
	Within 28 days prior C1D1	Day 1	Day 15		
**Sign ICF**[1]	X				
**Demography**	X				
**MedicalHistory**	X				
**Histologic/cytologic confirmation**[2]	X				
**Previous surgery, radiotherapy and treatments**	X				
**ECG**	X				
**Inclusion/Non-inclusion criteria**	X				
**Pregnancy Testing**[3]	X				
**Patient number**[4]	X				
**Dispense Trifluridine/Tipiracil**[5]		X			
**Dispense Panitumumab**[5]		X	X		
**Efficacy measurements**					

**Tumor Measurements**	**X**[6]			**X**[7]	**X**[7]
**Survival Status**		→	→	→	→
**Safety Measurements**					
**Physical Examination**	**X**	**X**[9]	**X**[9]		**X**
**ECOG**	**X**[8]	**X**[9]	**X**[9]		**X**
**Height**	**X**				
**12-lead ECG**	**X**				
**Vital signs & Weight**	**X**	**X**[9]	**X**[9]		**X**
**Haematology**[10]	**X**	**X**[12]			**X**
**Biochemistry**[11]	**X**	**X**[12]			**X**
**Urinalysis**	**X**				
**CEA, CA19-9**[13]	**X**			**X**	**X**
**Plasma Exploratory Biomarkers**[14]	**X**			**X**	**X**
**Concomitant treatments**	**X**	→	→	→	**X**
**AE Assessment**[15]	**X**	→	→	→	**X**
**Other measurements**					

Table 9. Study Assessment

Notes:

1. Sign Informed Consent Form (ICF): Written informed consent must be obtained prior to the performance of any study procedure.
2. Histologic/Cytological confirmation: Histological or cytological confirmation of metastatic adenocarcinoma of the colon or rectum, *RAS* wt, prior to administration of study drugs.
3. Pregnancy Testing: performed with serum βHCG test within 14 days prior to C1D1. Note: More frequent pregnancy assessments should be performed if required by local law.
4. Once patient has signed ICF, connection to the IWRS for patient's registration and eCRF patient number.
5. Study Medication: Trifluridine / Tipiracil will be administered twice daily (BID) on Days 1 to 5 and 8 to 12 of each treatment cycle. Panitumumab on day 1 and day 15 of each cycle.
6. Tumour Measurement at Screening Period: Tumour evaluation performed prior to patient ICF signature may be used if the date of the evaluation is within 28 days prior to randomization.
7. Tumour Measurement at Treatment Period: Perform tumour assessment based on general practices and standard of care of the site every 8 week ± 7 days from C1D1.
8. ECOG Performance Status: Patient's performance status must remain 0 or 1 during the screening
9. Physical examination, ECOG Performance Status, Vital signs and Weight have to be performed at screening and on Day 1 and on Day 15 pre-dose study drugs administration of each cycle. Prior to administering study drug, verify that patients with toxicities have met resumption criteria.
10. Haematology (including: haemoglobin, haematocrit, Platelet Count, white blood cell [WBC] count, absolute neutrophil count [ANC]) has to be performed at screening and within 72 hours prior to Day 1 study drug administration of each cycle. Prior to starting subsequent cycles, verify that patients with toxicities have met resumption criteria prior to administering study drug.
11. Biochemistry including: glucose, blood urea nitrogen (BUN), creatinine or creatinine clearance, sodium, potassium, calcium, magnesium, chloride, total bilirubin with fractionation into direct and indirect (if total bilirubin elevated), alkaline phosphatase, aspartate aminotransferase [AST, or serum glutamic oxalo-acetic transaminase, SGOT], alanine aminotransferase [ALT, or serum glutamic-pyruvic transaminase, SGPT]) and lactate dehydrogenase (LDH) has to be performed at screening and within 72 hours prior to Day 1 study drug administration of each cycle.
12. Procedures (haematology and biochemistry) for the first cycle have not to be repeated if performed as baseline (screening) within 7 days prior to C1D1. Prior to starting subsequent cycles, verify that patients with toxicities have met resumption criteria prior to administering study drug.
13. CEA (Carcinoembryonic antigen) and CA19-9(Carbohydrate Antigen 19-9) have to be performed during the screening period, every 8 weeks ± 7 days and at the End of Treatment.
14. Blood samples for plasma biomarker analysis have to be collected during the screening period, every 8 weeks ± 7 days and at the end of treatment.
15. AE Assessment: AE will be recorded from the first dose of study medication until the withdrawal visit. Any AE that occur prior to the first dose of study medication should be recorded as Medical History except events associated with any procedure/condition required by the study protocol: procedure (exercise test, MRI, etc.), change or withdrawal of previous/concomitant treatment relating to the conditions of the protocol, or a product other than the study drug, taken as part of the protocol.

Note: Assessment Windows: A window of +/-3 days is allowable for study procedures, as long as the proper order is maintained.

5. RESPONSE EVALUATION

Response must be evaluated through repetition of the CT or MRI scan of chest, abdomen and pelvis every 8 weeks from treatment start. Response will be codified by the Investigator according to the RECIST (Response Evaluation Criteria In Solid Tumors) guidelines version 1.1, summarized below.

5.1. Measurability of tumor lesions

At the baseline evaluation, the lesions will be defined as follows:

**measurable**:	Lesions which can be accurately measured on at least one dimension (the longest diameter must be recorded) and found to be ≥10 mm with CT scan or ≥ 20 mm with conventional techniques Lymph node found to be ≥ 15 mm in short axis with CT scan.<br><br>Note: All tumor measurements must be recorded in millimeters (or decimal fractions of centimeters)
**non-measurable**:	Lesions that are measurable but small (largest Diameter<10 mm with CT scan or <20 mm with conventional techniques or pathological lymph nodes with ≥ 10 to <15 mm short axis).<br><br>Bone lesions, leptomeningeal disease, ascites, pleural/pericardial effusions, lymphangitis of skin or lung, and abdominal masses or organomegaly (that cannot be followed by CT or MRI).<br><br>Lesions that are located in previously irradiates areas

All measurements must be made using a centimeter rule or gauge. All the baseline evaluations must be performed as close to the treatment start date as possible, and in all cases no more than 4 weeks to randomization.

5.2. Identification of "target" and "non-target" lesions

All measurable lesions up to a maximum of 2 lesions per organ and 5 lesions in total, representative of all involved organs, should be identified as target lesions and recorded and measured at baseline assessment. The target lesions should be selected on the basis of their size (lesions with the longest diameter), trying to represent all the involved organs. In addition, target lesions should be those that lend themselves to reproducible repeated measurements. It may be the case that, on occasion, the largest lesion does not lend itself to reproducible measurement in which circumstance the next largest lesion which can be measured reproducibly should be selected. The sum of the baseline diameters (longest for

non-nodal lesions, short axis for nodal lesions) of all target lesions will be calculated, reported in the eCRF, and used as reference for defining the objective response. All other lesions are identified as non-target lesions and must also be recorded at baseline assessment. Measurement of these lesions is not required, but the presence, absence, or the eventual unequivocal progression of each of them must be noted during the follow-up, at each scheduled restaging

5.3 Evaluation of target lesion response

Complete response (CR):	disappearance of all the target lesions. Any pathological lymph nodes (whether target or non-target) must have reduction in short axis to <10mm.
Partial Response (PR):	reduction of at least 30% in the sum of diameters of the target lesions, compared to the baseline evaluation.
Progression (PD):	increase of at least 20% in the sum of diameters of target lesions and an absolute increase of at least 5 mm, compared to the lowest sum recorded since the start of the treatment, or the appearance of one or more new lesions.
Stable disease (SD):	neither a sufficient reduction to be defined as PR, nor a sufficient increase to be defined as PD

5.4. Evaluation of non-target lesion response

Complete Response (CR):	Disappearance of all non-target lesions. All lymph nodes must be non-pathological in size(<10 mm short axis)
Non Complete Response / Non Progression (CR/non-PD):	Persistence of one or more target lesions
Progression (PD):	Appearance of one or more new lesions or indisputable, clear progression of an existing non-target lesion.

5.5. Evaluation of best overall response

The best overall response is the best response recorded from the start of treatment until disease progression/recurrence (taking the lowest measurement recorded since the start of treatment as reference for the progression).

**Target Lesions**	**Non-Target Lesions**	**New Lesions**	**RECIST Response**
CR	CR	No	**CR**

CR	non-CR/non-	No	**PR**
CR	Not evaluated	No	**PR**
PR	CR o non-CR/non-PD or not evaluated	No	**PR**
SD	CR o non-CR/non-PD or not evaluated	No	**SD**
Not all evaluated	CR o non-CR/non-PD	No	**Not evaluable**
PD	Any	Yes or No	**PD**
Any	PD	Yes or No	**PD**
Any	Any	Yes	**PD**

An overall deterioration in the state of health leading to a suspension of the treatment without evidence of progression will be defined as a "symptomatic worsening". Patients who suspend treatment due to symptomatic worsening will be considered as non-responding

5.6. Reporting of results

All patients included in the study must be assessed for response to treatment. Each patient will be assigned one of the following categories: 1) complete response, 2) partial response, 3) stable disease, 4)progressive disease, 5) early death from malignant disease, 6) early death from toxicity, 7) early death because of other cause, or 8) unknown (not assessable, insufficient data).All patients will be included in the analysis of the response rate. Patients in response categories 4-8 will be considered as failing to respond to treatment.

6. BIOMARKERS

6.1 Blood Sample Collection and analysis

Patients should provide written informed consent to obtain plasma blood samples (at least 20 mL in EDTA tubes) at screening, tumor assessment time points and End of Treatment.
Plasma samples will be stored at -80 °C at local sites and subsequently will be shipped to the Sponsor.

6.2Tumor Tissue Sample Collection

Tumor Tissue Sample will be collected at baseline and subsequently will be shipped to the Sponsor

7. SAFETY INSTRUCTIONS AND GUIDANCE

7.1 Warning and management of specific toxicities

PANITUMUMAB

Summary of the Safety Profile

Based on an analysis of mCRC clinical trial patients receiving panitumumab monotherapy and in combination with chemotherapy (n = 2224), adverse reactions occurring in ≥ 20% of patients were gastrointestinal disorders (diarrhea [46%], nausea [39%], vomiting [26%], abdominal pain [23%], and constipation [23%]);

Hypersensitivity Reactions

Hypersensitivity reactions have been reported, including a fatal case of angioedema that occurred more than 24 hours after the infusion. Depending on the severity (eg, presence of bronchospasm, edema, angioedema, hypotension, need for parenteral medication, or anaphylaxis) and/or persistence of hypersensitivity reactions, permanently discontinue panitumumab.

Dermatologic and Soft Tissue Toxicity

Skin and subcutaneous tissue disorders, a pharmacologic effect observed with epidermal growth factor receptor (EGFR) inhibitors, were frequently reported (approximately 93% in patients across the monotherapy mCRC clinical trials [N = 1052]).It is recommended that patients wear sunscreen and hats and limit sun exposure while receiving panitumumab as sunlight can exacerbate any skin reactions that may occur. Patients who develop dermatologic or soft tissue toxicities while receiving panitumumab should be monitored for the development of inflammatory or infectious sequelae. Life-threatening and fatal infectious complications, including events of necrotizing fasciitis and/or sepsis, have been observed in patients treated with panitumumab. Rare cases of Stevens-Johnson syndrome and toxic epidermal necrolysis have been reported in patients treated with panitumumab in the post-marketing setting.
Stop or discontinue panitumumab for dermatologic or soft tissue toxicity associated with severe or life-threatening inflammatory or infectious complications.

Other Warnings and Precautions

Proactive skin treatment including skin moisturizer, sun screen (sun protection factor[SPF] > 15 ultraviolet A [UVA] and ultraviolet B [UVB]), topical steroid cream(not stronger than 1% hydrocortisone) and an oral antibiotic (eg, doxycycline), as prescribed by the physician, are useful in the management of skin toxicities. Subjects should be advised to apply moisturizer and sunscreen to face, hands, feet, neck, back, and chest every morning during treatment and, if any contraindications, to apply the topical steroid to face, hands, feet, neck, back, and chest every night. Treatment of skin

reactions should be based on severity and may include a moisturizer, sun screen (SPF >15 UVA and UVB), and topical steroid cream (not stronger than 1% hydrocortisone) applied to affected areas, and/or oral antibiotics, as prescribed by the physician.

Pulmonary Toxicity

Fatal and non-fatal cases of interstitial lung disease (ILD) have been observed in patients treated with EGFR inhibitors including panitumumab. In the event of acute onset or worsening of pulmonary symptoms, panitumumab therapy should be interrupted and a prompt investigation of these symptoms should occur. If ILD is confirmed, panitumumab should be permanently discontinued and the patient should be treated appropriately. In patients with a history of interstitial pneumonitis or pulmonary fibrosis or evidence of interstitial pneumonitis or pulmonary fibrosis, the benefits of therapy with panitumumab versus the risk of pulmonary complications must be carefully considered.

Laboratory Tests

Electrolyte Disturbances/Monitoring

Progressively decreasing serum magnesium levels leading to severe hypomagnesemia have been observed in some patients. Patients should be monitored for hypomagnesemia and accompanying hypocalcemia prior to initiating panitumumab treatment, and periodically during panitumumab treatment and for up to 8 weeks after the completion of treatment. Magnesium repletion is recommended, as appropriate.

Other electrolyte disturbances, including hypokalemia, have also been observed. Repletion of these electrolytes is also recommended, as appropriate.

Trifluridine-Tipiracil

Myelosuppressive toxicities

In the RECOURSE trial, the incidence of Grade ≥3 neutropenia was 38% in patients treated with Trifluridine/Tipiracil, the incidence of febrile neutropenia was 4%. In the event of Grade 4 neutropenia (absolute neutrophil count [ANC] <0.5 x 10^9/L) within a treatment cycle, the dose of Trifluridine/Tipiracil should be held until the ANC increases to ≥1.5 x 10^9/L(see section 4.3.2; Table 6-7). If the delay is longer than 1 week, the dose of Trifluridine/Tipiracil in the next cycle should be reduced by 5 mg/m2 per dose from the previous dose level. If febrile neutropenia occurs, the dose of Trifluridine/Tipiracil should be held until the episode of febrile neutropenia is completely resolved, and the drug dose in the next cycle should be reduced by 5 mg/m2 per dose from the previous dose level (see Table 8). Prophylactic administration of G-CSF is recommended to prevent the development of

febrile neutropenia, according to current guidelines (J. Klastersky et al 2016, ESMO guidelines). In the RECOURSE study G-CSF support was used in 9.4% of patients

Anemia

Red blood cell transfusion is the recommended treatment for patients with symptomatic anemia due to myelosuppressive chemotherapy, and the use of transfusions should also be considered for asymptomatic patients with anemia who have underlying comorbidities.

Thrombocytopenia

For platelet counts <0.5 x 10^9/L, the dose of Trifluridine/Tipiracil should be held until the platelet counts increase to ≥75 x 10^9/L (see section 4.3.2; Table 6- Table 7). If the delay is longer than 1 week, the dose of Trifluridine/Tipiracil in the next cycle should be reduced by 5 mg/m^2 per dose from the previous dose level. Prophylactic platelet transfusions are recommended at a threshold platelet count of 10 x 10^9/L for patients with solid tumors who have chemotherapy-induced thrombocytopenia.

Non-hematologic AEs

In the RECOURSE trial the Trifluridine/Tipiracil group had higher rates of Grade ≥3 nausea (2% vs. 1%), vomiting (2% vs. < 1%), and diarrhea (3% vs. < 1%). However, there were no clinically meaningful differences between the treatment groups in the percentage of patients with other non-hematologic AEs, including decreased appetite, stomatitis, and hand-foot syndrome. For Grade ≥3 non-hematologic AEs, the dose of Trifluridine/Tipiracil should be held until resolution of the AE to Grade 0 or 1 (see section 4.3.2; Table 6-7). With the exception of Grade 3 nausea, vomiting, or diarrhea that is controlled by medication, the dose of Trifluridine/Tipiracil in the next cycle should be reduced by 5 mg/m2 per dose from the previous dose level for Grade ≥3 non-hematologic AEs (Table 3).

7.2 Adverse Events and Laboratory Abnormalities

7.2.1 Clinical Adverse Events (AEs)

According to the International Conference of Harmonization (ICH), an AE is any untoward medical occurrence in a patient or clinical investigation subject administered a pharmaceutical product and which does not necessarily have a causal relationship with this treatment. An AE can therefore be any unfavorable and unintended sign [including an abnormal laboratory finding], symptom, or disease temporally associated with the use of a medicinal (investigational) product, whether or not considered related to the medicinal (investigational) product. Pre-existing conditions worsening during thestudy are to be reported as AEs.

Intensity

Intensity of all AEs will be graded according to the NCI CTCAE v.5.0 on a five-point scale (Grade 1 to 5) and reported in detail on the CRF.

AEs not listed on the CTCAE should be graded as follows:

- Grade 1: Mild Discomfort noticed but no disruption of normal daily activity
- Grade 2: Moderate Discomfort sufficient to reduce or affect daily activity; no treatment or medical intervention is indicated although this could improve the overall well-being or symptoms of the patient
- Grade 3: Severe Inability to work or perform normal daily activity; treatment or medical intervention is indicated in order to improve the overall wellbeing or symptoms; delaying the onset of treatment is not putting the survival of the patient at direct risk
- Grade 4: Life threatening/disabling. An immediate threat to life or leading to a permanent mental or physical conditions that prevents work or performing normal daily activities; treatment or medical intervention is required in order to maintain survival
- Grade 5: AE resulting in death

7.2.2Drug AE relationship Adverse (drug) reaction (ADR)

The causality relationship of study drug to the AE will be assessed by the Investigator as either: *Yes* or *No*
If there is a reasonable suspected causal relationship to the study medication, i.e. there are facts (evidence) or arguments to suggest a causal relationship, drug-event relationship should be assessed as Yes.

The following criteria should be considered in order to assess the relationship as Yes:

- Reasonable temporal association with drug administration
- It may or may not have been produced by the patient's clinical state, environmental or toxic factors, or other modes of therapy administered to the patient
- Known response pattern to suspected drug
- Disappears or decreases on cessation or reduction in dose
- Reappears on re-challenge
- An **adverse (drug) reaction** (ADR) is a response to a medicinal product which is noxious and unintended. Response in this context means that a causal relationship between a medicinal product and an adverse event is at least a reasonable possibility. Adverse reactions may arise from use of the product within or outside the terms of the marketing authorization or from occupational exposure. Conditions of use outside the marketing authorization include overdose, misuse, abuse, and medication errors.

The following criteria should be considered in order to assess the relationship as No:

- It does not follow a reasonable temporal sequence from administration of the drug
- It may readily have been produced by the patient's clinical state, environmental or toxic factors, or other modes of therapy administered to the patient
- It does not follow a known pattern of response to the suspected drug
- It does not reappear or worsen when the drug is re-administered

7.2.3 Serious Adverse Events (SAE)

A SAE is any experience that suggests a significant hazard, contraindication, side effect or precaution. It is any AE that at any dose fulfills at least one of the following criteria:

- is fatal (results in death; NOTE: death is an outcome, not an event)

- is Life-Threatening (NOTE: the term "Life-Threatening" refers to an event in which the patient was at immediate risk of death at the time of the event; it does not refer to an event which could hypothetically have caused a death if it had been more severe).
- requires in-patient hospitalization or prolongation of existing hospitalization;
- results in persistent or significant disability/incapacity;
- is a congenital anomaly/birth defect;
- is another Important Medical Event (any important adverse events/reactions that is not immediately life-threatening or do not result in death or hospitalization, but may jeopardize the subject or may require medically significant or requires intervention to prevent one or other of the outcomes listed above).
- Examples of such events include, but are not limited to, intensive treatment in an emergency room or at home for allergic bronchospasm, blood dyscrasias or convulsions that do not result in hospitalization).

Note: The term sudden death should be used only when the cause is of a cardiac origin as per standard definition. The terms death and sudden death are clearly distinct and must not be used interchangeably. The study will comply with all local regulatory requirements and adhere to the full requirements of the ICH Guideline for Clinical Safety Data Management, Definitions and Standards for Expedited Reporting.

7.2.4 Serious adverse drug reaction (SADR)

A **Serious adverse drug reaction** (SADR) is an adverse reaction which results in death, is life-threatening, requires in-patient hospitalization or prolongation of existing hospitalization, results in persistent or significant disability or incapacity, or is a congenital anomaly/birth defect.
Medical and scientific judgment should be exercised in deciding whether other situations should be considered serious adverse reactions, such as important medical events that might not be immediately life threatening or result in death or hospitalization but might jeopardize the patient or might require intervention to prevent one of the other outcomes listed above.
Examples of such events are intensive treatment in an emergency room or at home for allergic bronchospasm, blood dyscrasias, or convulsions that do not result in hospitalization, or development of dependency or abuse.
Any suspected transmission via a medicinal product of an infectious agent is also considered a serious adverse reaction.

7.2.5 Special situations:

Overdose

An overdose is a significant variation above the recommended/scheduled dosage for a product. In this current trial an overdose of the study drugs is any dose higher than the dose specified in the Sections 4.3.1.

There is no known antidote available in case of Trifluridine/Tipiracil overdose. Overdose should be managed aggressively with close monitoring and administration of prophylactic and symptomatic therapies to prevent or correct potential side effects.

Abuse, misuse, occupational exposure

Misuse refers to situations where the medicinal product is intentionally and inappropriately used not in accordance with the authorized product information.

Abuse corresponds to the persistent or sporadic, intentional excessive use of a medicinal product, which is accompanied by harmful physical or psychological effects.

Occupational exposure refers to the exposure to a medicinal product, as a result of one's professional or non-professional occupation.

Medication errors

A medication error for this study is defined as any accidental incorrect administration of a medicinal product. The error may be related to the administration of a wrong medication, nature of the medication, route of administration, dosage or frequency of the treatment as specified in this protocol (including omission of one or more administrations).

- Medication errors with concomitant medication treatment will not be recorded in the CRF unless they result in an AE.
- Medication errors with the study medication that result in an AE will be captured as an AE in the CRF.
- Medication errors with the study medication that result in the omission of an administration, an incorrect dose (relative to that specified in this protocol), or the administration of more than the prescribed dose will be identified through the recording of study drug accountability data in the CRF.
- Medication errors with the study medication resulting in an overdose, incorrect route of administration, or administration with the incorrect study drug will be reported

Planned Hospitalization

A hospitalization planned by the subject prior to signing the informed consent form (ICF) is considered a therapeutic intervention and not the result of a new SAE and should be recorded as medical history. If the planned hospitalization or procedure is executed as planned, the record in the subject's medical history is considered complete. However, if the event/condition worsens during the trial, it must be reported as an AE.

7.3 Treatment and Follow-up of AEs

Please see section **7.1.** for general guidelines for management of specific toxicities.

A general principle is that differential diagnoses should be diligently evaluated according to standard medical practice. Non-inflammatory etiologies should be considered and appropriately treated.

The treatment and follow up of all the AEs should follow the indication provided below.

- For Related AEs, follow until one of the following occurs:
  - Resolved or improved to baseline;
  - Relationship is reassessed as unrelated;
  - Death;
  - Investigator confirms that no further improvement can be expected;
  - Clinical or safety data will no longer be collected, or final database closure.
- For Unrelated severe or life-threatening AEs, follow until one of the following occurs:
  - Resolved or improved to baseline;
  - Severity improved to Grade 2;
  - Death;
  - Investigator confirms that no further improvement can be expected;
  - Clinical or safety data will no longer be collected, or final database closure.

The final outcome of each AE must be recorded on the CRF.

7.4 Laboratory Test abnormalities

Laboratory test results will be recorded on the laboratory results form of the eCRF, or appear on electronically produced laboratory reports submitted directly from the central laboratory, if applicable. Any treatment-emergent abnormal laboratory result that is clinically significant, i.e., meeting one or more of the following conditions, should be recorded as a single diagnosis on the AE page in the eCRF:

- Accompanied by clinical symptoms

- Leading to a change in study medication (e.g. dose modification, interruption or permanent discontinuation)
- Requiring a change in concomitant therapy (e.g. addition of, interruption of, discontinuation of, or any other change in a concomitant medication, therapy or treatment)

This applies to any protocol and non-protocol specified safety and efficacy laboratory result from tests performed after the first dose of study medication, which falls outside the laboratory reference range and meets the clinical significance criteria.

7.4.1 Follow-up of Abnormal Laboratory Test Values

In the event of medically significant unexplained abnormal laboratory test values, the tests should be repeated and followed up until they have returned to the normal range and/or an adequate explanation of the abnormality is found. If a clear explanation is established it should be recorded on the eCRF.

7.5 Handling of Safety Parameters

7.5.1 Reporting of AEs

Information about all adverse events, whether volunteered by the patient, discovered by Investigator questioning, or detected through physical examination, laboratory test or other means, will be collected on the Adverse Event CRF page, documented in the patient's medical records, and followed as appropriate.

The NCI CTC-AE (Version 5.0) will be used to evaluate the clinical safety of the treatment in this study. Patients will be assessed for AEs at each clinical visit and as necessary throughout the study.

7.5.2 Reporting of SAEs (immediately reportable)

Any clinical AE, or abnormal laboratory test value assessed as serious (as defined above), pregnancy case, occurred during the course of this study from the enrolment visit (start of study screening procedures), including long term follow-up, must be reported to Sponsor and within *one* working day of the Investigator becoming aware of the event (expedited reporting).

The Investigator must complete the SAE form and forward it to the SAE Responsible person:

**Emergency contact**	Prof. Fortunato Ciardiello	0815666729

	Dr E. Martinelli, Dr T. Troiani	0815666628

In addition, report of Adverse Events will be done, according to current local law, to local Health Authorities.

From the first administration of study drugs, all SAEs must be reported. Related SAEs MUST be collected and reported regardless of the time elapsed from the last study drugs administration, even if the study has been closed. Suspected Unexpected Serious Adverse Reactions (SUSARs) are reported to Investigators at each site and associated IRB/IEC when the following conditions occur:

- The event must be a SAE
- There must be a certain probability that the event is an adverse reaction from the administered drugs
- The adverse reaction must be unexpected, that is to say, not foreseen in the Investigator's Brochure of study drugs. When all patients at a particular site are off treatment as defined by the protocol:
  - only individual SUSAR reports originating in that particular trial will be forwarded to the site and associated Insitutional Review Board (IRB) / Independent Ethics Committee (IEC) on an expedited basis
  - individual SUSARs considered to be a significant safety issue and/or which result in recommending a change to the ICF, will be reported in an expedited manner to all Investigators and IRBs/IECs
- SUSAR reports originating from other trials using the same study drugs will be provided as six monthly SUSAR Reports to Investigators and IRBs/IECs where long-term follow-up studies are carried out, unless they are considered significant

Unrelated SAEs must be collected and reported during the study and for up to five years after the last dose of study medication and every 3 months for long-term follow up.

This study adheres to the definition and reporting requirements of ICH Guideline for Clinical Safety Data Management, Definitions and Standards for Expedited Reporting.

7.5.3 Pregnancy

Female patients must be instructed immediately inform the investigator if they become pregnant during the study. The investigator should report all pregnancies within 24 hours to the Sponsor and, using the Clinical Trial Pregnancy Reporting Form. The investigator should counsel the patient, discuss the risks of continuing with the pregnancy and the possible effects on the fetus.

Pregnancies occurring up to 6 months after the completion of the study medication must also be reported to the Investigator. Pregnancies occurring in the partner of a male patient participating in the study

should be reported to the investigator and the sponsor. The partner should be counseled, the risks of continuing the pregnancy discussed, as well as the possible effects on the fetus. Monitoring of the patient should continue until conclusion of the pregnancy.

7.5.4 Safety data exchange requirement

Investigators should forward all SAEs and SUSARS to the Sponsor as soon as possible, but no later than 1 business day; pregnancy and lactation reports must be sent within 7days.
The Sponsor will provide Amgen with a copy of any SAE report received by the Investigators. Furthermore a copy of SUSARS must be sent to Amgen at the time of regulatory submission.
The Sponsor will also provide Amgen with a yearly Safety Report and with a copy of any other communication. Aggregate reports, containing safety data generated during the course of the study are to be sent to the Regulatory Authorities and to Amgen by the sponsor.
The final study report to be sent to Amgen no later than one calendar year from study completion.

The Sponsor will provide Servier with a copy of any SAE report received by the Investigators. Furthermore, a copy of SUSARS must be sent to Servier at the time of regulatory submission.
The Sponsor will also provide Servier with a yearly Safety Report and with a copy of any other communication. Aggregate reports, containing safety data generated during the course of the study are to be sent to the Regulatory Authorities and to Servier by the sponsor
The final study report to be sent to Servier no later than one calendar year from study completion

8. STATISTICAL CONSIDERATIONS

8.1 Sample size calculation

This is a randomized phase II, open label, two arm study, evaluating the efficacy of Trifluridine-Tipiracil in combination with panitumumab as third line therapy, after a first line containing an anti-EGFR agent panitumumab or cetuximab.
The study is designed to have 80% power to detect a hazard ratio for progression of 0.56 (a 44% reduction in risk) in the panitumumab+ Trifluridine-Tipiracil group as compared with the Trifluridine-Tipiracil group, with a two-sided type I error rate of 0.1. Given the treatment assignment ratio of 1:1 (Trifluridine-Tipiracil: panitumumab + Trifluridine-Tipiracil), we calculate that at least 74 events (progression) in 112 subjects (56 in the control group and 56 in the treatment group) would be required for the primary analysis.

8.2 Analysis Populations

Baseline is defined as the last valid visit or day before the treatment start. The following populations are defined for this study:

All Enrolled Set

All screened subjects who have been enrolled.

Intention To Treat (ITT) set

All enrolled patients that start the treatment will be considered the Intention-To-Treat population (ITT).

Safety Set

The subset of patient of the ITT population receiving at least one dose of the study medication will define the Safety Population (SP).

8.3 Statistical analysis

The median period of follow-up will be calculated for the entire study cohort according to the reverse Kaplan–Meier method. Distributions of time-to-event variables will be estimated with the use of the Kaplan–Meier product-limit method. The stratified log-rank test will be used as the primary analysis for comparison of treatment groups. Cox proportional-hazards modeling will be also performed as supportive analyses. Subgroup analyses of progression-free survival will be performed by means of an interaction test to determine the consistency of the treatment effect according to key baseline characteristics. Overall survival will be analyzed with the same methods used for the analysis of progression-free survival.

The objective response rate and the incidence of adverse events in the different groups will be compared with the use of the chi-square test for heterogeneity.

All statistical tests will be two-sided, and P values of 0.05 or less will be considered to indicate statistical significance. Odds ratios and 95% confidence intervals will be estimated with a logistic-regression model, and hazard ratios and 95% confidence intervals will be estimated with a Cox proportional hazards model.

<u>Data handling</u>

ORR and its association with prognostic factors will be investigated using the chi-square test and a logistic regression model will be used to study interrelationship among factors.

Safety and tolerability will be assessed in terms of AEs, laboratory data, ECG data, vital signs and weight, which will be collected for all patients. AEs (both in terms of MedDRA preferred terms and CTCAE grade), laboratory data, ECG data, vital signs data and weight will be listed individually by the patient and summarized by treatment received. Worst grade for each toxicity will be reported according to their relationship to study drugs. Treatment discontinuation due to AEs will also be described according to the treatment exposure.

Treatment duration, dose intensity, dose modification and discontinuation will be analyzed in each arm. Due to the small sample size, statistical analysis of **biomarkers data** will be conducted with the aim of hypothesis generation. A complete description of data will be done. For biomarkers that might change over time as a consequence of treatment, levels before and after treatment will be compared with appropriate statistical tests, based on the type of data. Repeated measurement ANOVA model will be used to test differences along time.

Correlation with Outcomes will be evaluated with univariate regression models. P values ≤ 0.05 will be considered significant, and no adjustment is planned for multiple comparisons due to the exploratory nature of the analysis of the secondary endpoints.

9. DATA COLLECTION AND MANAGEMENT

9.1 Data confidentiality

Information about study subjects will be kept confidential and managed under the applicable laws and regulations. The data collection system for this study uses built-in security features to encrypt all data for transmission in both directions, preventing unauthorized access to confidential participant information. Access to the system will be controlled by a sequence of individually assigned user identification codes and passwords, made available only to authorized personnel who have completed prerequisite training.

9.2 Site monitoring

Before study initiation, at a site initiation visit or at an investigator's meeting, Sponsor personnel will review the protocol and CRFs with the investigators and their staff. During the study, the field monitor will visit the site regularly to check the completeness of patient records, the accuracy of entries on the CRFs, the adherence to the protocol to Good Clinical Practice, the progress of enrolment, and to ensure that study treatment is being stored, dispensed, and accounted according to specifications. Key study personnel must be available to assist the field monitor during these visits. The investigator must maintain source documents for each patient in the study, consisting of case and visit notes (hospital or clinic medical records) containing demographic and medical information, laboratory data, electrocardiograms and the results of any other tests or assessments. All information recorded on CRFs must be traceable to source documents in the patient's file. The investigator must also keep the original signed informed consent form (a signed copy is given to the patient). The investigator must give the monitor access to all relevant source documents to confirm their consistency with the CRF entries. The Sponsor monitoring standards require full verification for the presence of informed consent, adherence to the inclusion/exclusion criteria and documentation of SAEs.

9.3 Data collection

This study will use an Electronic Data Capture (EDC) system (eClinical platform provided by Clinical Research Technology). The designated investigator staff will enter the data required by the protocol into the Electronic Case Report Forms (eCRF). The eCRFs have been built using eClinical platform provided by Clinical Research Technology, a fully validated secure web-enabled software that conforms to Food and Drug Administration (FDA) requirements. Investigator site staff will not be given access to the EDC system until they have been trained. Automatic validation programs check for data discrepancies in the eCRFs allow modification or verification of the entered data by the investigator staff. The Principal Investigator is responsible for assuring that the data entered into eCRF is complete, accurate, and that entry and updates are performed in a timely manner.

9.4 Database management and quality control

The Sponsor personnel (or designated contract research organization [CRO]) will review the data entered by investigational staff for completeness and accuracy. Electronic data queries stating the nature of the problem and requesting clarification will be created for discrepancies and missing values and sent to the investigational site via the EDC system. Designated investigator site staff is required to respond promptly to queries and to make any necessary changes to the data. Concomitant treatments entered into the database will be coded using the WHO Drug Reference List, which employs the Anatomical Therapeutic Chemical classification system. Medical history/current medical conditions and adverse events will be coded using the Medical dictionary for regulatory activities (MedDRA) terminology. The occurrence of any protocol violations will be determined. After the data have been verified to be complete and accurate, the database will be declared locked. Authorization is required prior to making any database changes to locked data, by joint written agreement between the Biostatistics and Data Management and the Sponsor.

10. ETHICAL CONSIDERATION

10.1 Regulatory and ethical compliance

This clinical study was designed, shall be implemented and reported in accordance with the ICH Harmonized Tripartite Guidelines for Good Clinical Practice, with applicable local regulations (including European Directive 2001/20/EC), and with the ethical principles laid down in the Declaration of Helsinki.

10.2 Responsibilities of the investigator and IEC

The protocol and the proposed informed consent form must be reviewed and approved by Independent Ethics Committee (IEC) of all participating centers before study start.

10.3 Informed consent procedures

Eligible patients may only be included in the study after providing written (witnessed, where required by law or regulation), IEC-approved informed. Informed consent must be obtained before conducting any study-specific procedures (i.e. all of the procedures described in the protocol). The process of obtaining informed consent should be documented in the patient source documents. The date when a subject’s Informed Consent was actually obtained will be captured in their CRFs.

10.4 Publication of study protocol and results

The key design elements of this protocol will be posted in the publicly accessible database clinicaltrialsregister.eu. The Investigators assure that results of this study will be submitted for publication and reported in scientific meetings.

10.5 Study documentation, record keeping and retention of documents

Each participating site will maintain appropriate medical and research records for this trial, in compliance with Section 4.9 of the ICH E6 GCP, and regulatory and institutional requirements for the protection of confidentiality of subjects. Each site will permit authorized representatives of the sponsor and regulatory agencies to examine (and when required by applicable law, to copy) clinical records for the purposes of quality assurance reviews, audits and evaluation of the study safety and progress. Source data are all information, original records of clinical findings, observations, or other activities in a clinical trial necessary for the reconstruction and evaluation of the trial. Examples of these original documents and data records include, but are not limited to, hospital records, clinical and office charts, laboratory notes, memoranda, subjects' diaries or evaluation checklists, pharmacy dispensing records, recorded data from automated instruments, copies or transcriptions certified after verification as being accurate and complete, microfiches, photographic negatives, microfilm or magnetic media, x-rays, and subject files and records kept at the pharmacy, at the laboratories, and medico-technical departments involved in the clinical trial. Data collection is the responsibility of the clinical trial staff at the site under the supervision of the site Principal Investigator. The study CRF is the primary data collection instrument for the study. The investigator should ensure the accuracy, completeness, legibility, and timeliness of the data reported in the CRFs and all other required reports. Data reported on the CRF, that are derived from source documents, should be consistent with the source documents or the discrepancies should be explained. All data requested on the CRF must be recorded. Any missing data must be explained. For electronic CRFs an audit trail will be maintained by the system. The investigator/institution should maintain the trial documents as specified in Essential Documents for the Conduct of a Clinical Trial (ICH E6 Section 8) and as required by applicable regulations and/or guidelines. The investigator/institution should take measures to prevent accidental or premature destruction of these documents. Essential documents (written and electronic) should be retained for a period of not less than seven (7) years from the completion of the Clinical Trial unless Sponsor provides written permission to dispose of them or, requires their retention for an additional period of time because of applicable laws, regulations and/or guidelines

10.6 Confidentiality of study documents and patient records

The investigator must ensure anonymity of the patients; patients must not be identified by names in any documents submitted to the Sponsor. However, the Sponsor may have access to source data. Signed informed consent forms and patient enrolment log must be kept strictly confidential to enable patient identification at the site.

10.7 Audits and inspections

Source data/documents must be available to inspections by the Sponsor or designee or Health Authorities.

10.8 Financial disclosures

Financial disclosures should be provided by study personnel who is directly involved in the treatment or evaluation of patients at the site - prior to study start.

11. PROTOCOL ADHERENCE

Investigators ascertain they will apply due diligence to avoid protocol deviations. Under no circumstances should the investigator contact the Sponsor or its delegates, if any, monitoring the study to request approval of a protocol deviation, as no authorized deviations are permitted. If the investigator feels a protocol deviation would improve the conduct of the study this must be considered a protocol amendment, and unless such an amendment is agreed upon by the Sponsor and approved by the IEC it cannot be implemented.

11.1 Amendments to the protocol

Any change or addition to the protocol can only be made in a written protocol amendment that must be approved by the Sponsor, Health Authorities where required, and the IEC. Only amendments that are required for patient safety may be implemented prior to IEC approval. Notwithstanding the need for approval of formal protocol amendments, the investigator is expected to take any immediate action required for the safety of any patient included in this study, even if this action represents a deviation from the protocol.

12. ADMINISTRATIVE ASPECTS

The study is a non-profit investigator initiated trial not sponsored by the pharmaceutical company which produce the drug used in the trial.
Panitumumab will be provided by AMGEN, Trifluridine-Tipiracil by ServierItaly.
The promoter will provide an insurance policy to cover possible damages caused to patients participating in the trial.
Study protocol, patient information and informed consent will be submitted to Independent Ethical Committees and will only be started after their approval. Independent Ethical Committees will be periodically informed of study progress, safety and planned or premature end of the study.

12. 1 Investigational medical product

12.1.1 Formulation, Packaging and Labelling

Study drug packaging will bear a label with the identification required by local law, the protocol number, drug identification and dosage.

Drugs will be administered open label. Drugs will be required to be labelled as per local SOPs and regulations.

Medication labels will be in the local language and comply with the legal requirements of each country in which the study will be conducted.

12.1.2 Accountability, assessment of compliance and destruction of the drugs

Study treatments must be received by designated personnel at the study site, handled and stored safely and properly, and kept in a secured location to which only the Investigator and designated site personnel have access.

Upon receipt, the study drugs should be stored according to the instructions specified on the drug labels and Investigator's Brochure for drugs (Panitumumab and Trifludine-Tipiracil). Study medication is to be stored in a secure locked area while under the responsibility of the Investigator. Receipt and dispensing of study medication must be recorded by an authorized person at the Investigator's site.

Any unused study drugs can only be destroyed after being inspected and reconciled by the responsible Study Monitor unless study drug containers must be immediately destroyed as required for safety, or to meet local regulations (e.g., cytotoxics or biologics).

On-site destruction is allowed provided the following minimal standards are met:

- On-site disposal practices must not expose humans to risks from the drug.
- On-site disposal practices and procedures are in agreement with applicable laws and regulations, including any special requirements for controlled or hazardous substances.
- Written procedures for on-site disposal are available and followed. The procedures must be filed with the site's SOPs and a copy provided to the study sponsor upon request.
- Records are maintained that allow for traceability of each container, including the date disposed of, quantity disposed, and identification of the person disposing the containers. The method of disposal, i.e., incinerator, licensed sanitary landfill, or licensed waste disposal vendor must be documented.
- Accountability and disposal records are complete, updated, and available for the Monitor to review throughout the clinical trial period.

If conditions for destruction cannot be met the responsible Study Monitor will make arrangements for return of study drug.

It is the investigator's responsibility to arrange for disposal of all empty containers, provided that procedures for proper disposal have been established according to applicable state, local, and institutional guidelines and procedures, and provided that appropriate records of disposal are kept.

REFERENCES

Arnold D, Lueza B, Douillard JY, Peeters M, Lenz HJ, Venook A, Heinemann V, Van Cutsem E, Pignon JP, Tabernero J, Cervantes A, Ciardiello F. Prognostic nad predeictive value of primary tumour side in

patients with RAS wild-type metastatic colorectal cancer treated with chemotherapy and GFR directed antibodies in six randomized trials. Ann Oncol. 2017 Aug 1;28(8):1713-1729. doi: 10.1093/annonc/mdx175

Baba Y, Tamura T, Satoh Y, Gotou M, Sawada H, Ebara S, Shibuya K, Soeda J, Nakamura K.Mol Panitumumab interaction with TAS-102 leads to combinational anticancer effects via blocking of EGFR-mediated tumor response to trifluridine. Oncol. 2017 Aug;11(8):1065-1077. doi: 10.1002/1878-0261.12074. Epub 2017 May 30.

de Gramont, A. et al. Leucovorin and fluorouracil with or without oxaliplatin as first- line treatment in advanced colorectal cancer. J. Clin. Oncol. 18, 2938–2947 (2000).

Douillard J.Y. et al. Irinotecan combined with fluorouracil compared with fluorouracil alone as first-line treatment for metastatic colorectal cancer: a multicentre randomised trial. Lancet 355, 1041–1047 (2000).

Douillard,J.Y.et al. Randomized,phaseIIItrial of panitumumab with infusional fluorouracil, leucovorin, and oxaliplatin (FOLFOX4) versus FOLFOX4 alone as first-line treatment in patients with previously untreated metastatic colorectal cancer: the PRIME study. J. Clin. Oncol. 28, 4697–4705 (2010).

Falcone, A. et al. Phase III trial of infusional fluorouracil, leucovorin, oxaliplatin, and irinotecan (FOLFOXIRI) compared with infusional fluorouracil, leucovorin, and irinotecan (FOLFIRI) as first-line treatment for metastatic colorectal cancer: the GruppoOncologico Nord Ovest. J. Clin. Oncol. 25, 1670–1676 (2007).

Ferlay J, Soerjomataram I, Ervik M, et al. GLOBOCAN 2012 v1.0, Cancer Incidence and Mortality Worldwide: IARC Cancer Base No. 11. Lyon, France: International Agency for Research on Cancer, 2013

Grothey A, Van Cutsem E, Sobrero A, Siena S, Falcone A, Ychou M, Humblet Y, Bouché O, Mineur L, Barone C, Adenis A, Tabernero J, Yoshino T, Lenz HJ, Goldberg RM, Sargent DJ, Cihon F, Cupit L, Wagner A, Laurent D; CORRECT Study Group. Regorafenibmonotherapy for previously treated metastatic colorectal cancer (CORRECT): an international, multicentre, randomised, placebo-controlled, phase 3 trial. Lancet. 2013 Jan 26;381(9863):303-12

Hurwitz, H. et al. Bevacizumab plus irinotecan, fluorouracil, and leucovorin for metastatic colorectal cancer. N. Engl. J. Med. 350, 2335–2342 (2004).

Jeevan M Puthiamadathil and Benjamin A Weinberg. Emerging combination therapies for metastatic colorectal cancer – impact of trifluridine/tipiracil Cancer Manag Res. 2017; 9: 461–469.

J. Klastersky, J. de Naurois, K. Rolston, B. Rapoport, G. Maschmeyer, M. Aapro and J. Herrstedt Management of Febrile Neutropaenia: ESMO Clinical Practice GuidelinesAnnOncol (2016) 27 (suppl 5): v111-v118 (J. Klastersky et al 2016, ESMO guidelines)

Mayer RJ, Van Cutsem E, Falcone A, Yoshino T, Garcia-Carbonero R, Mizunuma N, Yamazaki K, Shimada Y, Tabernero J, Komatsu Y, Sobrero A, Boucher E, Peeters M, Tran B, Lenz HJ, Zaniboni A, Hochster H, Cleary JM, Prenen H, Benedetti F, Mizuguchi H, Makris L, Ito M, Ohtsu A; RECOURSE Study Group. Randomized trial of TAS-102 for refractory metastatic colorectal cancer. N Engl J Med. 2015 May 14;372(20):1909-19.

Mayer RJ, Van Cutsem E, Falcone A, Yoshino T, Garcia-Carbonero R, Mizunuma N, Yamazaki K, Shimada Y, Tabernero J, Komatsu Y, Sobrero A, Boucher E, Peeters M, Tran B, Lenz HJ, Zaniboni A, Hochster H, Cleary JM, Prenen H, Benedetti F, Mizuguchi H, Makris L, Ito M, Ohtsu A; RECOURSE

Study Group. Randomized trial of TAS-102 for refractory metastatic colorectal cancer. N Engl J Med. 2015 May 14;372(20):1909-19. doi: 10.1056/NEJMoa1414325.

Pietrantonio F, Perrone F, Biondani P, Maggi C, Lampis A, Bertan C, et al. Single agent panitumumab in KRAS wild-type metastatic colorectal cancer patients following cetuximab-based regimens: Clinical outcome and biomarkers of efficacy. Cancer BiolTher. 2013;14(12):1098–1103.

Santini D, Vincenzi B, Addeo R, Garufi C, Masi G, Scartozzi M, et al. Cetuximab rechallenge in metastatic colorectal cancer patients: how to come away from acquired resistance? Ann Oncol. 2012;23(9):2313–2318.

Schirripa M, Lenz HJ. Colorectal cancer: Overcoming resistance to anti-EGFR therapy - where do we stand? NatRevGastroenterolHepatol. 2016 May;13(5):258-9. doi: 10.1038/nrgastro.2016.52. Epub 2016 Mar 23.

Sforza V, Martinelli E, Ciardiello F, Gambardella V, Napolitano S, Martini G, Della Corte C, Cardone C, Ferrara ML, Reginelli A, Liguori G, Belli G, Troiani T. Mechanisms of resistance to anti-epidermal growth factor receptor inhibitors in metastatic colorectal cancer.
World J Gastroenterol. 2016 Jul 28;22(28):6345-61.

Takeshi Kato, Yoshinori Kagawa, Yoshito Komatsu, Eiji Oki, Takayuki Yoshino, Kentaro Yamazaki, Hirofumi Yasui, HironagaSatake, Kazunori Shibuya, Koji Oba, and Kensei Yamaguchi. A phase I/II study for panitumumab combined with TAS-102 in patients with *RAS* wild-type metastatic colorectal cancer (APOLLON study): Phase I results.
Journal of Clinical Oncology 2017 35:4_suppl, 770-770

Takeshi Kato, Yoshinori Kagawa, Yoshito Komatsu, Eiji Oki, Takayuki Yoshino, Kentaro Yamazaki, Journal of Clinical Oncology 35, no. 4_suppl (February 2017) 770-770.

Van Cutsem E. et al. Cetuximab and chemotherapy as initial treatment for metastatic colorectal cancer. N. Engl. J. Med. 360, 1408–1417 (2009).

Van Cutsem E, Tabernero J, Lakomy R et al. Addition of aflibercept to fluorouracil, leucovorin, and irinotecan improves survival in a phase III randomized trial in patients with metastatic colorectal cancer previously treated with an oxaliplatin- based regimen. J ClinOncol 2012; 30: 3499–3506.

Van Cutsem E, Cervantes A, Adam R, Sobrero A, Van Krieken JH, Aderka D, Aranda Aguilar E, Bardelli A, Benson A, Bodoky G, Ciardiello F, D'Hoore A, Diaz-Rubio E, Douillard JY, Ducreux M, Falcone A, Grothey A, Gruenberger T, Haustermans K, Heinemann V, Hoff P, Köhne CH, Labianca R, Laurent-Puig P, Ma B, Maughan T, Muro K, Normanno N, Österlund P, Oyen W, Papamichael D, Pentheroudakis G, Pfeiffer P, Price TJ, Punt C, Ricke J, Roth A, Salazar R, Scheithauer W, Schmoll HJ, Tabernero J, Taïeb J, Tejpar S, Wasan H, Yoshino T, Zaanan A, Arnold D. ESMO consensus guidelines for the management of patients with metastatic colorectal cancer. Ann Oncol. 2016 Aug;27(8):1386-422.

Wadlow RC, Hezel AF, Abrams TA, Blaszkowsky LS, Fuchs CS, Kulke MH, et al. Panitumumab in patients with KRAS wild-type colorectal cancer after progression on cetuximab. Oncologist. 2012;17(1):14. doi: 10.1634/theoncologist.2011-0452.

Yasutoshi Kuboki, Takayuki Yoshino, Takeshi Kato, Yoshinori Kagawa, Makio Gamoh, Hirofumi Yasui, Kentaro Yamazaki, Yoshito Komatsu, Hironaga Satake, Masahiro Goto, Hiroaki Tanioka, Eiji Oki, Masahito Kotaka, Akitaka Makiyama, Tadamichi Denda, Junpei Soeda, Kazunori Shibuya, Masaru Iwata, Koji Oba, Kensei Yamaguchi. APOLLON: A phase I/II study of panitumumab combined with TAS-102 in patients (pts) with RAS wild-type (wt) metastatic colorectal cancer (mCRC). J Clin Oncol 36, 2018 (suppl; abstr 3523)

Appendix 1:

Performance Status Criteria

**WHO Performance Status Scale**		**Karnofsky Performance Scale**	
**Grade**	Descriptions	**Percent**	Description
0	Normal activity. Fully active, able to carry on all pre-disease performance without restriction.	100	Normal, no complaints, no evidence of disease.
		90	Able to carry on normal activity; minor signs or symptoms of disease.
1	Symptoms, but ambulatory. Restricted in physically strenuous activity, but ambulatory and able to carry out work of a light or sedentary nature (e.g., light housework, office work)	80	Normal activity with effort; some signs or symptoms of disease.
		70	Cares for self, unable to carry on normal activity or to do active work.
2	In bed <50% of the time. Ambulatory and capable of all self-care, but unable to carry out any work activities. Up and about more than 50% of waking hours.	60	Requires occasional assistance, but is able to care for most of his/her needs.
		50	Requires considerable assistance and frequent medical care.
3	In bed >50% of the time. Capable of only limited self-care, confined in bed or chair more than 50% of waking hours.	40	Disabled, requires special care and assistance.
		30	Severely disabled, hospitalization indicated. Death not imminent.
4	100% bedridden. Completely disabled. Cannot carry on any self-care. Totallyconfined to bed or chair	20	Very sick, hospitalization indicated. Death not imminent.
		10	Moribund, fatal processes progressing rapidly.
5	Deceased.	0	Deceased.
