# Supplementary material for: Panitumumab Plus Trifluridine-Tipiracil as Anti–Epidermal Growth Factor Receptor Rechallenge Therapy for Refractory RAS Wild-Type Metastatic Colorectal Cancer: A Phase 2 Randomized Clinical Trial
Source: JAMA Oncol. 2023 May 18;9(7):966–70. doi: 10.1001/jamaoncol.2023.0655 (PMC10196928; doi:10.1001/jamaoncol.2023.0655)
Supplement: Supplement 2. — eMethods. Outcomes and Statistical Analysis eTable 1. Baseline Demographic and Disease Characteristics for the ITT Population at the Time of Randomization eTable 2. Treatment-Emergent Adverse Events eTable 3. Treatment-Emergent Adverse Events by Grade eFigure 1. Best Response in Study Population eFigure 2. Molecular Screening of the VELO Trial eFigure 3. Correlation of Molecular Alterations Detected on ctDNA by FoundationOne CDx Liquid Analysis With Clinical Data eFigure 4. Landscape of Molecular Alterations Detected by FoundationOne CDx Liquid Analysis in 24 Patients With RAS/BRAF WT at Baseline and at Progression of Disease [file jamaoncol-e230655-s002.pdf]

Supplementary Online Content

Napolitano S, De Falco V, Martini G, et al. Panitumumab plus trifluridine-tipiracil as anti–epidermal growth factor receptor rechallenge therapy for refractory *RAS* wild-type metastatic colorectal cancer: a phase 2 randomized clinical trial. *JAMA Oncol*. Published online May 18, 2023. doi:10.1001/jamaoncol.2023.0655

eMethods. Outcomes and Statistical Analysis
eTable 1. Baseline Demographic and Disease Characteristics for the ITT Population at the Time of Randomization
eTable 2. Treatment-Emergent Adverse Events
eTable 3. Treatment-Emergent Adverse Events by Grade
eFigure 1. Best Response in Study Population
eFigure 2. Molecular Screening of the VELO Trial
eFigure 3. Correlation of Molecular Alterations Detected on ctDNA by FoundationOne CDx Liquid Analysis With Clinical Data
eFigure 4. Landscape of Molecular Alterations Detected by FoundationOne CDx Liquid Analysis in 24 Patients With RAS/BRAF WT at Baseline and at Progression of Disease

This supplementary material has been provided by the authors to give readers additional information about their work.

© 2023 Napolitano S et al. *JAMA Oncology*.

eMethods. Outcomes and statistical analysis.

Outcomes

The primary endpoint of this randomized phase 2 trial was PFS, which was defined as the time from randomization to the earliest documented disease progression or death due to any cause. The median period of follow-up was calculated for the entire study cohort according to the reverse Kaplan–Meier method. Distributions of time-to-event variables was estimated with the use of the Kaplan–Meier product-limit method. The stratified log-rank test was used as the primary analysis for comparison of treatment groups. Cox proportional-hazards modeling was also performed as supportive analyses. The objective response rate and the incidence of adverse events in the different arms was compared with the use of the □ square test. All statistical tests were two-sided. Hazard ratios and 95% confidence intervals were estimated with the Cox proportional hazards model. Secondary endpoints were overall response rate (ORR), safety and overall survival (OS). Analysis of the primary endpoint as well as the secondary endpoints according to baseline *RAS/BRAF* mutation status was pre-specified. Additional exploratory endpoints included genomic analysis by NGS of plasma samples, that were collected at baseline and, if possible, at end of treatment in order to identify other ctDNA mutations, which could be related to cancer cell resistance mechanisms.

Statistical analysis

The study was designed to have 80% power to detect hazard ratio (HR) for progression of 0.56 (44% reduction in risk) in the panitumumab plus trifluridine/tipiracil arm as compared to the standard-of-care trifluridine/tipiracil arm, with a two-sided type I error rate of 0.1. Given the treatment assignment ratio of 1:1, 74 events (disease progression) would have been required for the primary analysis. Unfortunately, clinical trial activation in several centers as well as patient enrolment for this non-profit, academic study was severely affected by the COVID-19 pandemic. Therefore, the planned patient enrolment was not reached and in April 2022 it was decided to stop recruitment when 62 patients had entered the trial. Analysis was performed for the primary endpoint in September 2022, when 59 events (disease progression) occurred. However, as reported in Results, even with this reduction in events, the primary endpoint of the study was met with a reduction of the risk for PFS, that is better than the planned HR, that would have been originally required for considering the trial positive.

© 2023 Napolitano S et al. *JAMA Oncology*.

eTable 1: Baseline demographic and disease characteristics for the ITT population at the time of randomization.

ITT: intention to treat. Data are n (%), or median (range). ECOG Eastern Cooperative Oncology Group.

BASELINE CHARACTERISTICS	ARM A n=31 (%)	ARM B n=31 (%)	P Value
**Sex**			
**Male**	**17 (54.8)**	**19 (61.3)**	**0.61**
**Female**	**14 (45.2)**	**12 (38.7)**	
**Median Age**	**66 (32-82)**	**65 (39-81)**	
**Age Groups**			
**≤ 65**	**15 (48.4)**	**16 (51.6)**	**0.80**
**> 65**	**16 (51.6)**	**15 (48.4)**	
**ECOG PS**			
**0**	**22 (71.0)**	**21 (67.7)**	**0.78**
**1**	**9 (29.0)**	**10 (32.3)**	
**Site of primary tumor**			
**Right**	**4 (12.9)**	**3 (9.7)**	**0.71**
**Left**	**20 (64.5)**	**23 (74.2)**	
**Rectum**	**7 (22.6)**	**5 (16.1)**	
**First line anti-EGFR drug**			
**Panitumumab**	**23 (74.2)**	**21 (67.7)**	**0.58**
**Cetuximab**	**8 (25.8)**	**10 (32.3)**	
**Resected primary tumor**			
**Yes**	**25 (80.6)**	**26 (83.9)**	**0.74**
**No**	**6 (19.4)**	**5 (16.1)**	
**Synchronous metastases**			
**Synchronous**	**21 (67.7)**	**22 (71.0)**	**0.78**
**Metachronous**	**10 (32.3)**	**9 (29.0)**	
**Baseline ctDNA**			
***RAS* Wild-type**	**23 (74.2)**	**26 (83.9)**	**0.35**
***RAS* Mutated**	**8 (25.8)**	**5 (16.1)**	
***BRAFV600E* Wild-type**	**31 (100)**	**31 (100)**	
***BRAFV600E* Mutated**	**-**	**-**	

© 2023 Napolitano S et al. *JAMA Oncology*.

eTable 2. Treatment-emergent adverse events
Data are n (%). No treatment-related deaths occurred.

	ARM A n=31 (%)	ARM B n=31 (%)	P Value
**Adverse Event (AE) grade**			
**1-2**	**15 (48.4)**	**14 (45.2)**	**0.039**
**3-4**	**9 (29)**	**16 (51.6)**	
**NO**	**7 (22.6)**	**1 (3.2)**	
**Hematologic toxicities***			
**NO**	**14 (45.2)**	**15 (48.4)**	**0.80**
**YES**	**17 (54.8)**	**16 (51.6)**	
**Non-Hematologic toxicities***			
**NO**	**17 (54.8)**	**14 (45.2)**	**0.45**
**YES**	**14 (45.2)**	**17 (54.8)**	
**Skin toxicities**			
**NO**	**31 (100)**	**7 (22.6)**	**0.0001**
**YES**	**0 (0)**	**24 (77.4)**	
**Dose reduction**			
**NO**	**22 (71.0)**	**15 (48.4)**	**0.07**
**YES**	**9 (29.0)**	**16 (51.6)**	
**Median cycles**	**2 (1-10)**	**4 (1-26)**	

***Non-haematological toxicities: diarrhoea, hand-foot syndrome, nausea and stomatitis;**
***Haematological toxicities: anaemia, neutropenia and febrile neutropenia, decreased platelet count.**

eTable 3. Treatment-emergent adverse events by grade

	ARM A n=31 (%)		ARM B n=31 (%)	
	G1-G2 (%)	G3-G4 (%)	G1-G2 (%)	G3-G4 (%)
**Skin toxicities**				
**Rash**	-	-	14 (45)	6 (19)
**Dry skin**	-	-	7 (23)	-
**Nail disordes**	-	-	6 (19)	-
**Pruritus**	-	-	2 (6)	-
**Conjunctivitis**	-	-	1 (3)	-
**Stomatitis**	-	-	4 (13)	-
**HFS**	-	-	2 (6)	-
**Gastrointestinal disorders**				
**Diarrhea**	4 (13)	-	6 (19)	-
**Abdominal pain**	2 (6)	-	1 (3)	1 (3)
**Nausea**	5 (16)	-	6 (19)	-
**Vomiting**	7 (23)	-	3 (10)	1 (3)
**AST/ALT increase**	-	-	1 (3)	-
**Blood birilubin increase**	1 (3)	-	2 (6)	-
		-		-
**Lipase and/or amylase increase**	1 (3)		-	
**Anorexia**	3 (10)	-	1 (3)	-
**Hematologic adverse event**				
**Neutropenia**	7 (23)	8 (26)	5 (16)	8 (26)
**Anemia**	2 (6)	1 (3)	3 (10)	1 (3)
**Piastrinopenia**	2 (6)	-	3 (10)	1 (3)
**General disorders**				
**Asthenia**	8 (26)	-	11 (35)	2 (6)
**Hypomagnesemia**	-	-	2 (6)	2 (6)
**Hypocalcemia**	-	-	1 (3)	-
**Hypertension**	-	-	-	1 (3)

HFS: hand-foot syndrome; AST: aspartate amminotrasferase; ALT: alanine amminotrasferase.

© 2023 Napolitano S et al. *JAMA Oncology*.

eFigure 1. Best response in study population.

A. Best response in ITT population: P= 0.009. **B.** Best response in *RAS/BRAF* WT population: P= 0.025. **C.** Best response in *RAS/BRAF* mutated population: P= 0.51. **D.** PFS >6 months: ITT population P=0.016; *RAS/BRAF* WT population P= 0.047; *RAS/BRAF* mutated population P= 0.62. **E.** PFS >12 months: ITT population P=0.040; *RAS/BRAF* WT population P= 0.052; *RAS/BRAF* mutated population P= 1.00.

ITT: intention to treat. CR: complete response. PR: partial response. ORR: overall response rate. SD: stable disease. PD: progression disease. DCR: disease control rate. PFS: progression free survival.

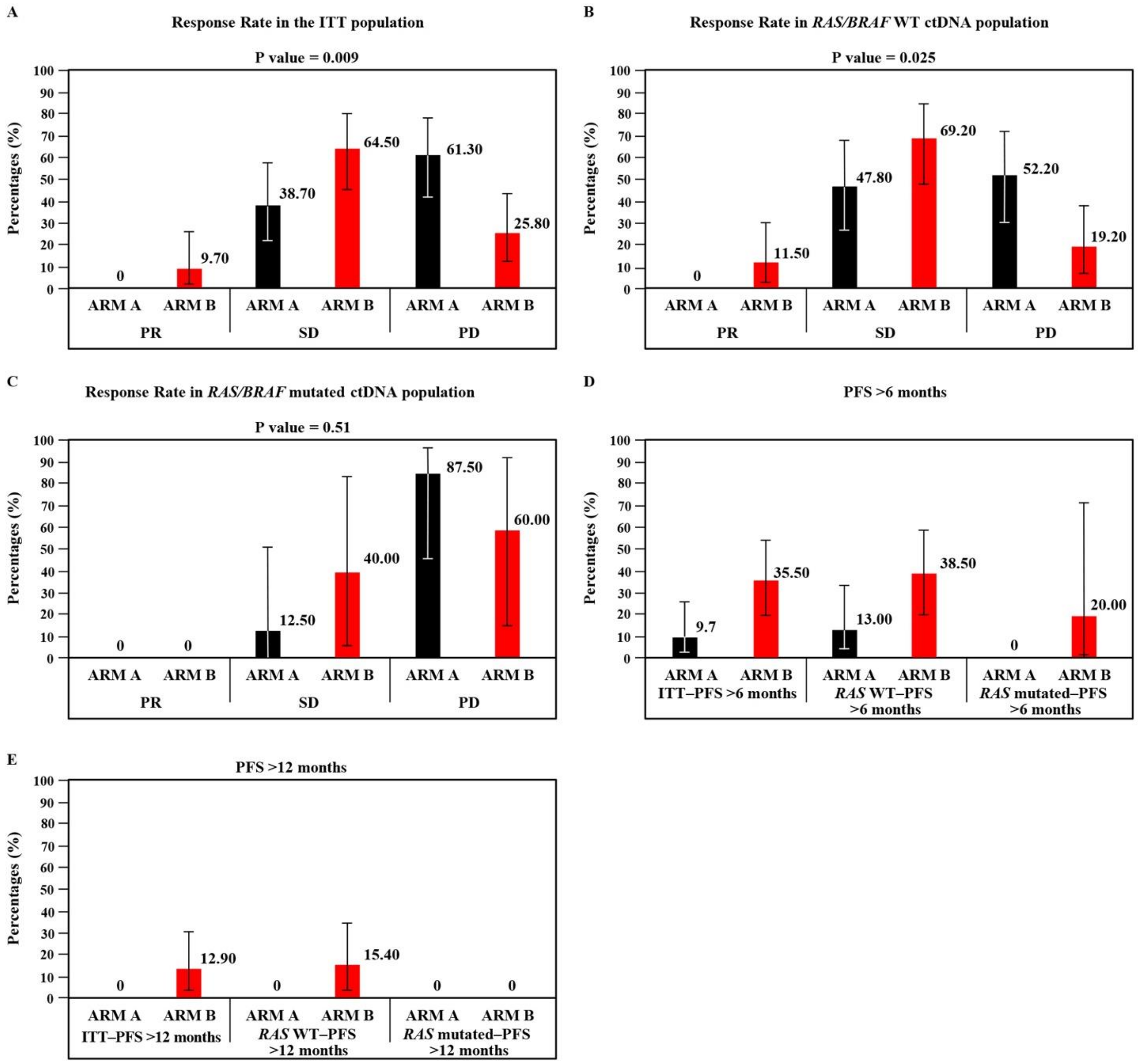

© 2023 Napolitano S et al. *JAMA Oncology*.

eFigure 2. Molecular screening of the VELO trial.

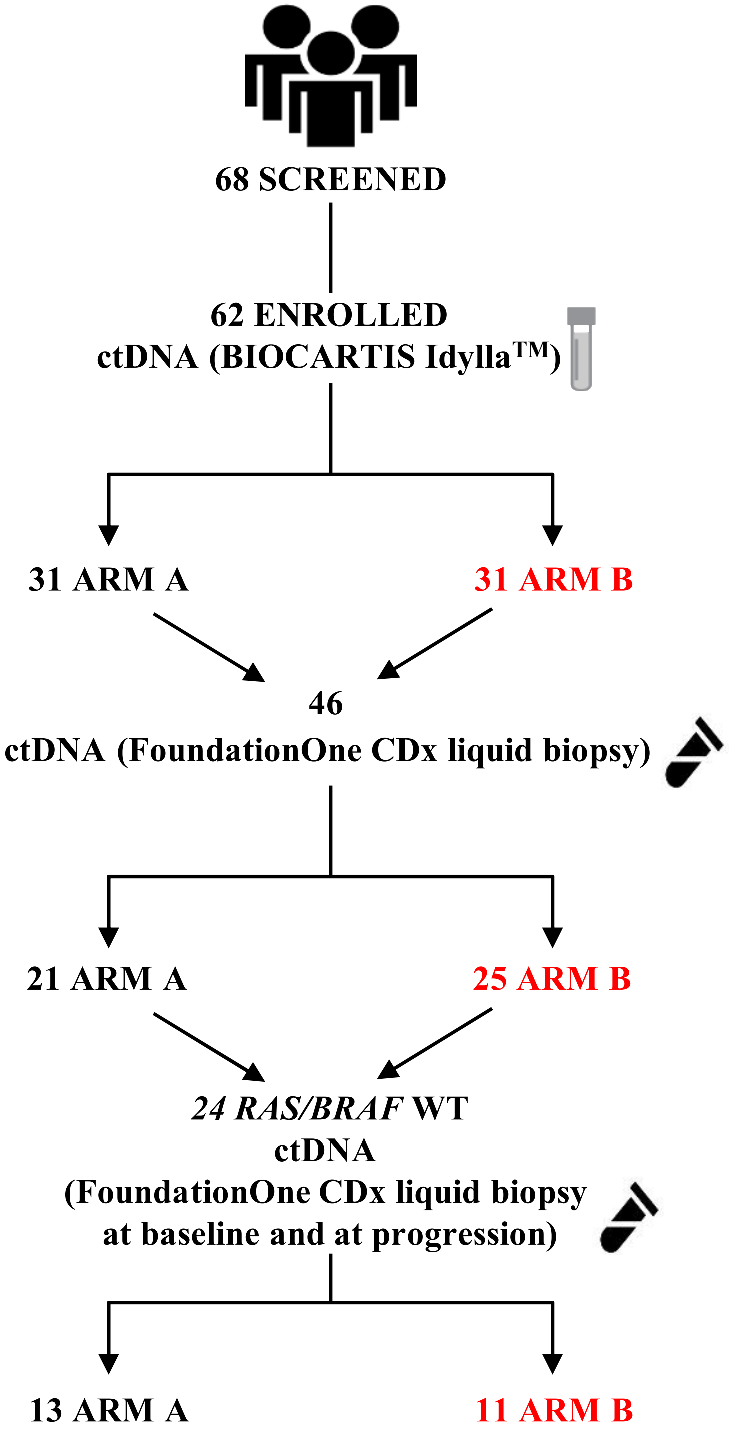

© 2023 Napolitano S et al. *JAMA Oncology*.

eFigure 3. Correlation of molecular alterations detected on ctDNA by FoundationOne CDx liquid analysis with clinical data.

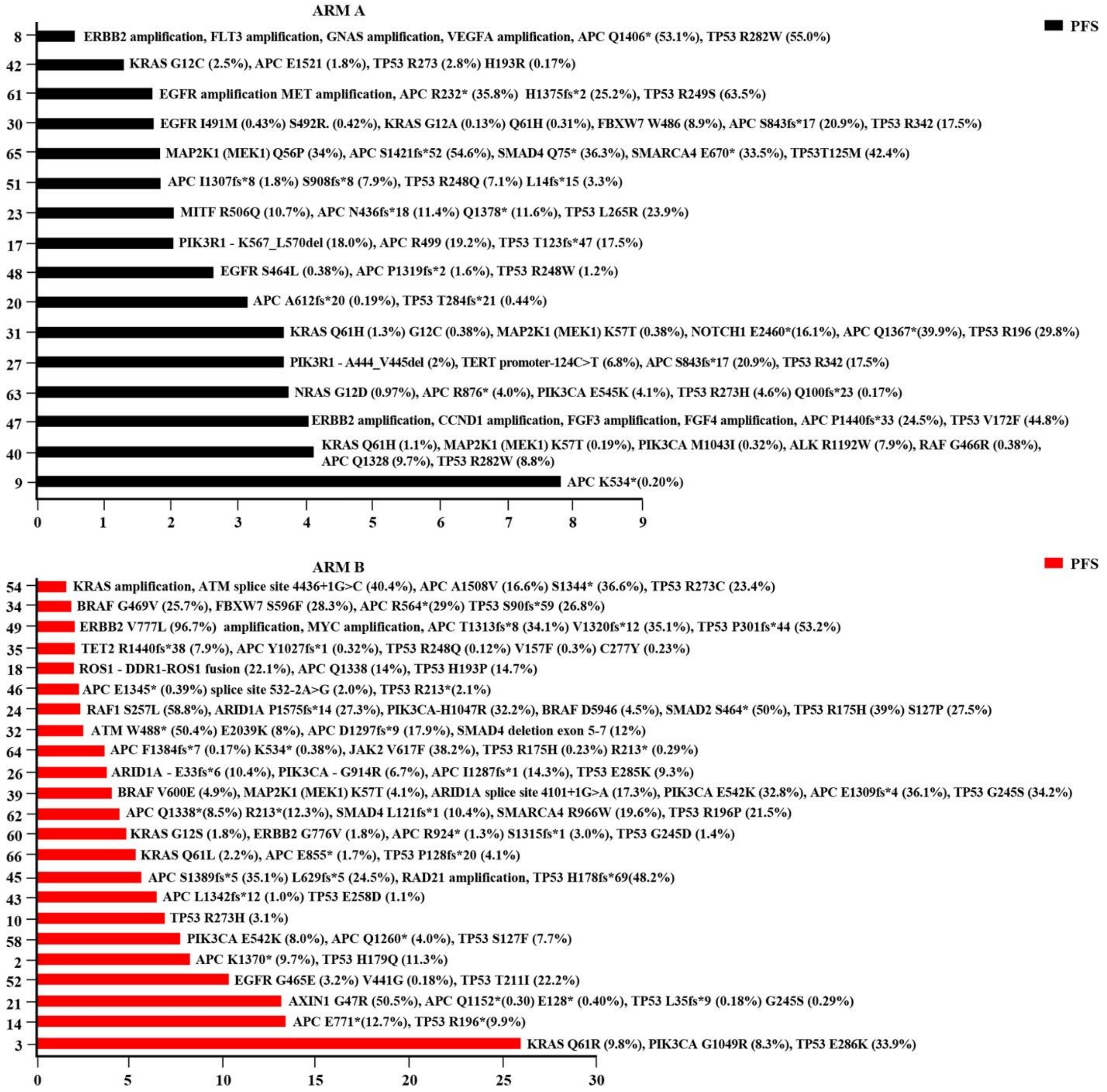

Each bar represents each patient with progression free survival time. Time on x-axys is time in months. On the y-axis every bar represents a single patient. Most relevant molecular alterations, detected before rechallenge treatment, are illustrated for individual patients.

© 2023 Napolitano S et al. *JAMA Oncology*.

eFigure 4. Landscape of molecular alterations detected by FoundationOne CDx liquid analysis in 24 RAS/BRAF WT patients at baseline and at progression of disease.

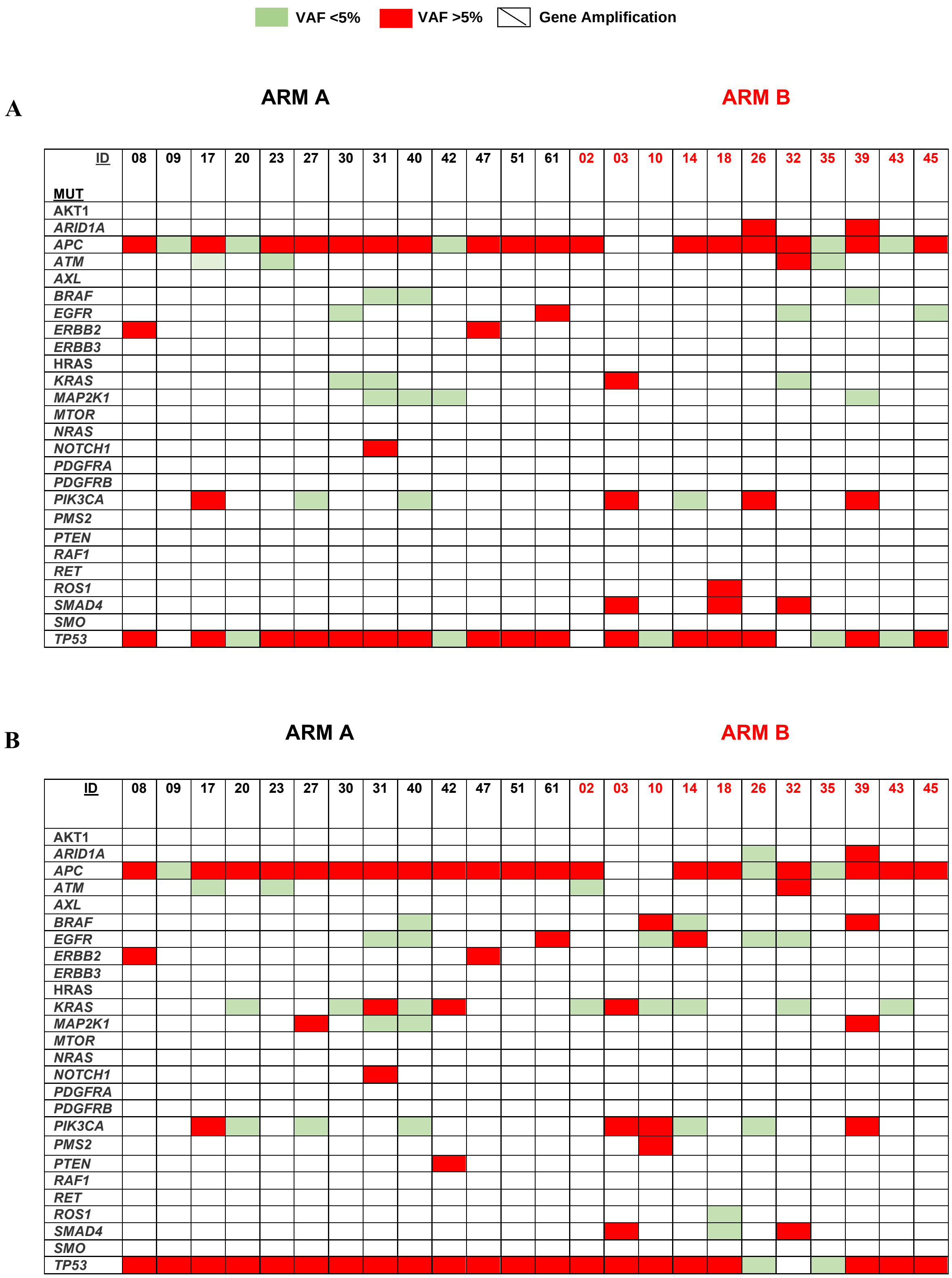

ctDNA samples were collected from 24 RAS/BRAF WT patients at baseline rechallenge and at progression disease and analyzed by FoundationOne CDx liquid test. **A**. The mutational status of the most frequently altered genes per patient and per treatment arm at rechallenge

© 2023 Napolitano S et al. *JAMA Oncology*.

baseline. **B.** The mutational status of the most frequently altered genes per patient and per treatment arm at progression disease.

Light green bar represents VAF < 5%. Red bar represents VAF >5%. Barred bar represents Gene Amplification.

VAF: variant allele fraction.

© 2023 Napolitano S et al. *JAMA Oncology*.
