# Supplementary material for: Panitumumab Plus Trifluridine-Tipiracil as Anti–Epidermal Growth Factor Receptor Rechallenge Therapy for Refractory RAS Wild-Type Metastatic Colorectal Cancer: A Phase 2 Randomized Clinical Trial
Source: JAMA Oncol. 2023 May 18;9(7):966–70. doi: 10.1001/jamaoncol.2023.0655 (PMC10196928; doi:10.1001/jamaoncol.2023.0655)
Supplement: Supplement 4. — Data Sharing Statement [file jamaoncol-e230655-s004.pdf]

Data Sharing Statement

Napolitano. Panitumumab Plus Trifluridine-Tipiracil as Anti-Epidermal Growth Factor Receptor Rechallenge Therapy for Refractory RAS Wild-Type Metastatic Colorectal Cancer. *JAMA Oncol*. Published May 18, 2023. doi:10.1001/jamaoncol.2023.0655

Data
Data available: Yes
Data types: Deidentified participant data
How to access data: Researchers can request access to de- identified individual patient-level data through the clinical study data request "Vanvitelli" platform. Data will be provided with an accompanying dictionary for the correct definition of each item reported in the clinical study documentation. If necessary, further details on sharing clinical information will be available by sending an email to trialsclinici@unicampania.it and to the corresponding author.
When available: With publication

Supporting Documents
Document types: None

Additional Information
Who can access the data: researchers whose proposed usa of the data has been approved
Types of analyses: for a specified purpose
Mechanisms of data availability: with investigator support
